# Context‐Sensitive Conscious Interpretation and Layer‐5 Pyramidal Neurons in Multistable Perception

**DOI:** 10.1002/brb3.70393

**Published:** 2025-03-04

**Authors:** Talis Bachmann

**Affiliations:** ^1^ Institute of Psychology University of Tartu Tartu Estonia

**Keywords:** conscious interpretation, layer‐5 pyramidal neuron, perception

## Abstract

**Introduction:**

There appears to be a fundamental difference between the two ways of how an object becomes perceptually experienced. One occurs when preconscious object‐specifying sensory data processing crosses a certain threshold so that sensory constituents of object depiction become consciously experienced. The other occurs when the already consciously experienced sensory features of the object become interpreted as belonging to a certain visual object category. Surprisingly, experimental facts about neural markers of conscious access gathered so far do not allow us to distinguish mechanisms responsible for these two varieties.

**Methods:**

A cortical multicompartment layer‐5 pyramidal neuron‐based generic processing model is presented in order to conceptualize a possible mechanistic solution for the explanatory cul‐de‐sac. To support the argument, a review of pertinent research is compiled as associated with data from studies where physically invariant perceptual stimuli have underwent alternative interpretation(s) by the brain.

**Results:**

Recent developments in the newly emerging field of cellular psycho(physio)logy are introduced, offering a hypothetical solution for distinguishing the mechanisms subserving sensory content experience and conscious interpretation.

**Conclusion:**

The multicompartment single cell‐based mechanistic approach to brain process correlates of conscious perception appears to have an added value beyond the traditional inter‐areal connectivity‐based theoretical stances.

## Graphical abstract

Context‐sensitive multicompartment layer‐5 pyramidal neurons are well suited for carrying out the disambiguating interpretive function for physically invariant ambiguous images.

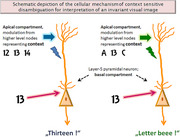

## 1 Introduction

In mainstream brain‐based consciousness science, contrastive analysis has stood out as the “golden standard” of research for quite a long time (Boly et al. 2017; Frith et al. 1999; Koch et al. 2016; Crick and Koch 1990). In order to reveal the neural correlates of consciousness (NCC), experiments have been designed to compare brain processes measured in conditions where a subject consciously perceives certain content and when (s)he does not perceive that content. The measured neural differences between conscious versus not conscious conditions are taken as an NCC (i.e., as markers of consciousness). However, for varied reasons, this contrastive analysis is by no means straightforward (Aru et al. 2012; Klein et al. 2020; Lepauvre and Melloni 2021; Navajas et al. 2014; Paßler 2023; Pitts et al. 2014). One of the possible reasons why the progress in finding “true” NCC has been slow is because conscious perception involves different levels or aspects. Correspondingly, we have to consider NCCs for different aspects of perception.

The early level (or aspect) of perception (re)presents the set of basic *featural attributes* pertaining to objects and scenes. For example, we experience dots, lines, edges, shapes, surfaces, textures, colors, motions, and so forth—all arranged in specific ways characteristic to each specific actual environment. Some parts of the observable layout of features when grouped together form *perceptual objects* governed by rules of perceptual organization and neurally represented in hierarchically organized multiple brain areas (Bracci and Op de Beeck 2023; Köhler and Adams 1958; Treisman 1987; Van Geert and Wagemans 2024; Wagemans 2015; Wagemans et al. 2012). The set of features of which an object consists of specifies the sensory‐perceptual level of direct experience. However, objects belong to certain categories, each carrying specific cognitive meaning and behavioral‐adaptive affordance (“what it is; what can I expect from it; what can be done with it”) (Hansen 2024; Palmeri and Gauthier 2004). Thus, in order to experience the meaning/affordance of objects, to understand them, the formally organized feature content has to be *interpreted* in a certain way—this is a distinct aspect/level of perceptual experience intermingled with higher cognitive processing. We should ask whether consciousness of sensory content is seeing, knowing, or both, and how it happens (Lamme 2018). NCCs have been sought for all of the above‐listed aspects (reviews: Förster et al. 2020; Kim and Blake 2005; Koch 2004; Northoff and Lamme 2020; Promet and Bachmann 2024; Yaron et al. 2022). The tasks used for finding NCC for perceptual content have used target stimuli specified at the feature‐, visual object‐, and interpretation levels, but unfortunately without much success in distinguishing and specifying with which aspect the extracted NCC goes and whether the NCC is universal for all aspects (Bachmann and Aru 2023).

The above‐noted indiscriminate results on perceptual NCCs may stem from the prevalent; however implicit, assumption that for the perceptual content there is one universal brain mechanism subserving conscious access. Regardless of which kind of target stimuli and tasks we use, up to a certain stage of processing the target remains unconscious and as soon as the “conscious access threshold” is crossed thanks to the workings of this mechanism, the target becomes consciously experienced. A two‐stage scenario is adopted: unconscious processing of stimulus content is followed by conscious‐level processing of content. However, there is a somewhat more nuanced situation here. Instead of the one‐step account of conscious access, a two‐step account seems viable. First, there is conscious access to the sensory‐perceptual content (feature‐level content becomes phenomenally represented). Let us call this step‐1 conscious access. Second, a cognitive interpretation of the already consciously experienced sensory‐perceptual representation *adds* to the feature‐level content of consciousness (interpretation does not replace step‐1 conscious contents, it supplements it with itself.)

It is possible to argue that we always subjectively perceive interpreted scenes, and that we never experience just “features” without interpretation. However, there are a couple of reasons why the additional, interpretive step seems necessary. Suppose a visual noise field is shown without any well‐organized Gestalt involved. Initially it appears as a meaningless spatially dispersed mass of tiny local contrast increments (i.e., step‐1 has been carried out for actual sensory signals), sooner or later some part of these local increments spontaneously appears to form some object or configuration. Moreover, the additional step in forming conscious experience becomes especially obvious when we observe ambiguous images. The same set of spatially distributed local elements, such as edges, lines, and dots become to be perceived either in one or the other interpreted version. This occurs even without saccadic or pursuit eye movements when eyes are fixated on an ambiguous image so that the change in sensory input is minimal, if any. Let us call this step‐2 conscious access. Concomitantly, as Bachmann and Aru (2023) argue, we are allowed to speak about two varieties of NCC for perception—threshold‐NCC (NCCthr) marking step‐1 access and interpretation‐NCC (NCCint) marking step‐2 access. An obvious reason why this possibility seems to be often overlooked rests on the typical one‐to‐one correspondence between sensory content and its cognitive interpretation at the categorical (or ecological affordance) level. This one‐to‐one correspondence characterizes the vast majority of objects and perceptual events in most of the everyday situations. Step‐1 and step‐2 processes are typically so tightly coupled that their distinction is well hidden for subjective observation unless special cases of sensory signals are used and/or intricate experimental designs applied to disentangle the hidden stages. It could be expected that if multiple‐interpretable, ambiguous figures (e.g., invariant sensory content allowing more than one object interpretation) are used as the target stimuli in NCC research then we will finally distill NCCint. However, the results of many studies carried out by combining various behavioral experimental paradigms with brain imaging do not show that there are differences between NCCthr and NCCint (reviews: Bachmann and Aru 2023; Boly et al. 2017; Northoff and Lamme 2020).

The contents of consciousness are multiaspect. We directly experience the world at least at the physical features level content, wholistic objects level content, and abstract semantic level of content. Conscious experience is singular, with the just mentioned aspects amalgamated in this singularity, but its aspects bear differences in emphasis. This is possible due to persistent changes in the between‐aspects (attentional or Gestalt selection) focus in the stream of consciousness. When we experience an interpreted object, both the feel of interpreted *features* and the *interpretation* are present. However, certain specific sets of features can be associated with alternative sets of neural codes of interpretation.

The NCC research conducted so far that finds no distinct differences in the results for NCCthr and NCCint has been carried out almost exclusively by recording and imaging methods provided by functional magnetic resonance imaging (fMRI), electro‐encephalography (EEG), magneto‐encephalography (MEG), local field potential(s) (LFP), and occasionally on neurological patients with single‐cell recordings. But there must be NCC differences. After all, every different cognitive state must have a corresponding neural state; if the neural states were identical, the cognitive experience would be identical. This puzzle awaits to be solved if we want to have a powerful enough conceptualization of the NCC.

Also, the theoretical explanations of NCC markers have been predominantly at the large network/population level systems or microcircuits. Perhaps, then, some new recording and measurement methods as well as new theoretical paradigms different from the already established ones could be helpful in the quest for specific NCCs for NCCthr and NCCint as subaspects of conscious experience. Furthermore, the new theoretical approaches must be valid and appropriate for explaining the key principles and functions of the process of cognitive interpretation of sensory data. Later on, we will describe one such novel approach—cellular psychology founded on the concept of the cortical layer‐5 pyramidal neuron and its functioning mechanisms (review: Phillips et al. 2024).

Conscious interpretation, including in the case of ambiguous figures owes considerably to top‐down processing originating from the frontal cortex and having an effect on a relatively lower level where sensory/perceptual representations are located (Faivre et al. 2019; Ishizu and Zeki 2014; Mao et al. 2020; Pitts et al. 2008; Sen et al. 2020; Watanabe 2021; Weilnhammer et al. 2021). The role of top‐down processes in conscious interpretation comes to the fore especially in circumstances where context steers object disambiguation (Biderman et al. 2020; Klink et al. 2012; Tal et al. 2024). These considerations allow us to admit that the newly emerging paradigm of cellular psychology, exemplified by the dendritic integration theory and apical amplification mechanism (Aru et al. 2020a; Bachmann et al. 2020; Phillips 2023; Phillips et al. 2024) may become useful in the search for the putative NCCint. The core of these approaches presupposes modulation of the activity of perceptual content carrying context‐sensitive pyramidal neurons by top‐down and other types of presynaptic input. Importantly, apart from the traditional integrate‐and‐fire point neuron concept adopted in most of the well‐established theories, this mechanism is based on the *concept of multicompartment neuron* with different signal integration zones within one nerve cell. Distinct from the point‐neuron concept, the multicompartment neuron allows for a mechanistically important elementary processing unit where invariance of sensory signals in the (peri)somatic part of the cell membrane can combine with variance in the top‐down controlled modulatory signals in the other parts of the membrane. In part 2 let us have a closer look at this paradigm.

Before giving you the specifics of the multicompartment neuron approach, two qualifications are necessary. First, it would be naïve to believe that one nerve cell—even though it has multiple autonomous subcomponents—could bear responsibility of being the very “seat” of rich conscious experience. It is a matter of circuits of microcircuits instead where, I believe, layer‐5 cells play a central mechanistic part, but do not exhaust the list of all necessary mechanisms. There are several recent models of how to combine neurobiological multicompartment‐neuron mechanisms and neurophysiological mesoscale as well as more global processing. For example, the levels of processing model describing the neural hierarchy for consciousness (Almeida 2022), thalamocortical critical synchronous bursting model (Munn, Müller, Aru et al. 2023), and model of cellular neuromodulation effects on global brain‐wide functions (Shine et al. 2021). Second, the present paper remains deliberately agnostic on whether the multicompartment neuron is just a prerequisite correlate of conscious perception (NCCpr) or its direct correlate (NCC) (Aru et al. 2012). This is because some of the regimens of brain action accompanying unconscious states (e.g., generalized epileptic seizures and NREM sleep) also involve layer‐5 burst firing as a result of inter‐compartment activity coupling. Future studies are expected to specify bursting modes for conscious and unconscious states.

## 2 Materials and Methods

### 2.1 The Cellular‐Level Mechanism of Context‐Sensitive Modulation

The general blueprint of cellular‐level contextual modulation was presented by Larkum (2013) and Phillips et al. (2024) while more specific accounts were exemplified by two familiarly resemblant conceptualizations—dendritic integration theory (Aru et al. 2020a; Bachmann et al. 2020; Storm et al. 2024) and the context‐sensitive apical amplification mechanism (Marvan et al. 2021; Phillips 2017, 2023).

Specific physical information about objects, events, and scenes is processed by cortical nerve cells tuned to their “trigger content” such as oriented edges and lines, color, motion, shape, frequency pattern of sound, and so forth (Gazzaniga et al. 2019; Werner and Chalupa 2004). These cortical neurons receive bottom‐up input signals, form a networked hierarchy of representational complexity and are specialized for sensory content representation; they populate areas back in the cortex (occipital, temporal, parietal lobes). Another important function of cortical neurons in order to be cognitively apt is context sensitivity without which adequate adaptation to environmental challenges would be questionable. There is a special type of cortical neuron capable of fulfilling both functions, encoding specific perceptual content and mediating context effects: layer‐5 pyramidal neurons (L5p) with cell bodies (soma) in the 5th cortical layer and the apical trunk extending up to layer 1 of the cortex. Somatic and perisomatic dendrites receive input signals on specific sensory information transmitted by bottom‐up afferent pathways. These signals when sent to a set of L5p neurons’ (peri)somatic dendrites uniquely specify the featural content of the object that is the source of these signals. This kind of local dendritic innervation is independent of the higher cognitive effects. This zone of the L5p membrane featuring (peri)somatic dendrites we call the *basal compartment*. Apical dendrites close to the tuft in layer 1 specify the *apical compartment* of the L5p cell membrane. Presynaptic input to the apical compartment by and large originates from relatively higher cortical areas and mediates top‐down modulation of L5p activity. This input can originate from different higher‐level sources and is capable of carrying effects from different cognitively represented contexts. When a stimulus object defined by a set of its feature‐representing L5p is immersed in a current scene context, the bottom‐up signals to the basal cell compartment are invariant to different context, but the apical top‐down signals vary depending on what is the cognitive context. Therefore, the apical compartment is well capable of enabling contextual effects such as attention, conceptual meaning, object‐category effects, biased interpretation effects, and so forth.

**FIGURE 1:**
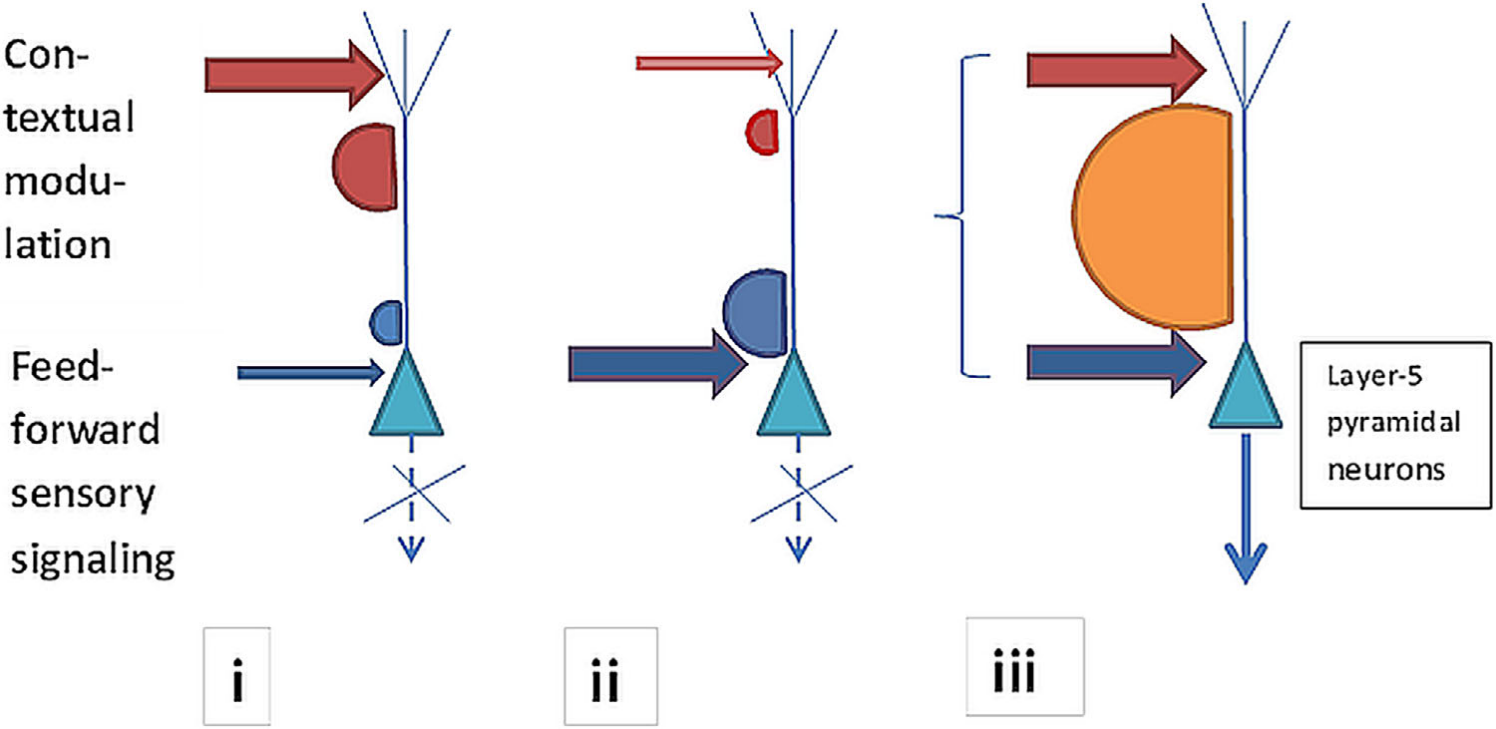
The L5p neuron contributes to integrative neural activity of the brain when there is sufficient simultaneous activity by apical and basal (perisomatic) spikes to contextual and sensory input, respectively (iii). Basal and apical coupling is necessary for cognition‐dependent perceptual capabilities subserved by the mass of L5p sending their action potentials (APs) to multiple brain areas. If basal activity is relatively weak/asynchronous (i) or if apical activity is relatively weak/asynchronous (ii), no sufficient mass of APs is produced by the set of L5p neurons. (Figure reproduced with permission from Bachmann and Hudetz (2014), It is time to combine the two main traditions in the research on the neural correlates of consciousness: C = L × D. Frontiers in Psychology, 5, 940.)

Importantly, rigorous and cerebrally wide‐spreading activity from L5p commences when basal and apical compartments receive sufficient simultaneous presynaptic input (Figure 1). The cell enters the burst mode of spiking (firing) facilitating active connectivity with other cortical areas and subcortical centers via action potentials sent to other neurons. Integration of activities or coupling of basal and apical compartments of L5p is mediated and facilitated by special mechanisms. First, the back‐propagating action potential activated Ca^2+^ (BAC) cellular firing mechanism. When sufficiently intense Na^+^ action potentials backpropagate from the basal compartment up along the dendrite simultaneously with sufficient Ca^2+^ spikes (and vice versa), somatic bursts or BAC firing occur. This exemplifies the active coupling of two integration zones of the cell membrane, one receptive for bottom‐up sensory signaling, the other receptive for top‐down context‐dependent input. This substantiates the augmented participation of information processed by this cell in higher integrative functions of the brain. Second, the effect of Ca^2+^ spikes on somatic firing depends on modulation of dendrites at the third (coupling) compartment of the neuron where dendrites are located at the trunk between soma and the apical zone (not shown in Figure 1). Basal compartment activity can be decoupled from apical compartment activity and contextual modulation by the input from non‐specific subcortical neurons directed at the coupling compartment; for example, this happens due to anesthesia—Suzuki and Larkum 2020.)

Burst mode of L5p activity allows for widespread delivery of information represented by that specific cell, including shipments to cortico‐cortical and thalamocortical loops responsible for consciousness (Aru et al. 2019; Bachmann 2021). In this process, feedforward input to the basal compartment carries information about ambient sensory content and almost by definition has to be associated with NCC mechanisms for phenomenal perceptual content of consciousness. The L5p neurons with this function are most likely located in the so‐called cortical hot zone in the more caudal lobes (Boly et al. 2017) as the corresponding receptive fields of these neurons are specialized for level *F* and level *O* content (as per subsequent pt 2.2). The top‐down presynaptic signals for contextual modulation and attentional control arrive at the apical compartment of L5p, which in terms of NCC are natural to associate with access consciousness and cognitive interpretation functions. I tend to agree with the view that the NCCthr varies gradually as a function of the relative share of the basal dendritic activity in producing burst firing of the mass of L5p representing phenomenal content. This process is likely marked by dedicated EEG signatures such as the amplitude and latency of VAN, PAN, or N170 (Almeida 2022; Filimonov et al. 2022; Förster et al. 2020; Jimenez et al. 2018). On the other hand, as conscious interpretation of the already activated sensory content is a higher‐level function mediated by the apical compartment directed modulation, the putative NCCint has to be associated with layer‐1 activities first of all. The candidate EEG marker for this would be P300b as a signature of the “conceptual” access to the phenomenal content (Almeida 2022; Filimonov et al. 2022; Förster et al. 2020; Jimenez et al. 2018); this is either by level‐*O* access, level‐*M* access, or both.

The elements of the L5p modulation mechanisms and the mechanisms of how this cellular level system works are multiple and complex (Phillips 2023; Shine et al. 2021; Capone et al. 2023; Munn, Müller, Medel et al. 2023; Gillon et al. 2024; Marvan and Phillips 2024). In addition to classical Na^+^ and Ca^2+^‐related synaptic EPSP mechanisms and HCN channels fundamentally involved in L5p action, there are varied cell membrane channel mechanisms and neuromodulation mechanisms dependent on NMDA receptors, AMPA receptors, glutamatergic modulation, GABA‐ergic effects, and ACh and aminergic neuromodulation (e.g., NA/NE, 5‐HT, DA), all impacting the functioning of context‐sensitive layer‐5 pyramidal neurons. Moreover, without understanding how a couple of different inhibitory or excitatory interneurons drive L5p and subdue its activity potential, we cannot understand cellular psychology and put forward or test respective hypotheses. Even the types of L5p context‐sensitive effects are multiple. For example, there are cellular‐level interactive micromechanisms capable of different types of cognitive information processing regulation: standard operations of excitation, inhibition, and disinhibition, but also amplification, attenuation, and disattenuation (Phillips et al. 2018; Phillips 2023). How, and to what extent, these operations are carried out depends on all the above‐listed participating elements. However, believably, the general overview of the L5p‐based contextual amplification and modulation mechanism given in this section is sufficient for an attempt to explain conscious interpretation as a step‐2 operation in conscious perception where neural signals necessary for interpretive experience combined with the neural signals necessary for experience of the physical features. Before coming to this hypothetical explanation later on, we must consider the general types of the context where contextual disambiguation may take place.

### 2.2 Context‐Sensitive Processing System and Examples of Contextual Phenomenal Effects

Perceptual interpretation of an articulated set of sensory signals as objects involves a myriad of hierarchically organized processes and is influenced by a multitude of factors (Bracci and Op de Beeck 2023). Already before the current more complex analyses, classic studies of cognitive psychology also described object recognition as a non‐unitary process. For example, Bruner and Minturn (1955, 1) wrote, “In perceiving, there are two analytically separable processes. The first is a process of spatio‐temporal‐qualitative organization that makes possible such phenomena as figure‐ground formation, the segregation of contours, the perception of plane surfaces, and so forth. The second process is usually called identification or recognition by which we are enabled to see an object *as* something. In the picturesque phrase of James, ‘There is a thingumbob again.’ Recognition or identification depends upon the presence in the perceptual field of a certain amount of perceptual segregation. In sum, if we were not able to segregate it from the background, we would not be able to identify an apple as an apple.”

For the purpose of the present paper, a somewhat simplified account of the levels of processing where sensory signals become interpreted in conscious object will be used. However, this simplified model does not violate the generally accepted view of how representations are stratified and processes unfold in the brain in order to lead to object recognition.

Ontologically, we have certain physical entities, physical objects O, characterized by certain physical attributes (objective features F) as a property of these objects. For perception, it is crucial to detect, discriminate, and recognize objects in order to be adaptively competent. This is aided by specialized nervous systems capable of representing features and objects and capable of making sense of their importance, including their rewarding capacity and existential dangerousness. The systems of representation are built as a hierarchical structure with different levels, *L*, of representational function and complexity.

The levels are as follows:

1. *S*—***stimulation*** *of receptors* signaling physical attributes, S, of the habitat pertaining to scenes of “things”;
2. *F*—activation of ***feature*** *‐encoding* units, F, in sensory‐cortical subsystems of the brain;
3. *O*—based on data from *F*, *representations of* ***objects***, O, are activated at next levels;
4. *M*—semantic processing of ***meaning***, M, by higher‐level cognitive categorization mechanisms.

Let us bear in mind that this linear sequence from *S* to *M* describes the hierarchy of levels with increasing abstraction of representations. The *processes* within and between levels involve both feedforward and feedback (top‐down, back‐propagating, recurrent) directions of between‐levels influence (Bar 2021; Bouvier and Treisman 2010; Lamme 2018; Long and Toppino 2004; Otten et al. 2017; Parr et al. 2019). Different structural levels of the brain subserve different stages of processing that unfold in time and use within‐ as well as between‐levels signaling via a multitude of neural pathways.

Taken from the introspective point of view, conscious perception presents subjects with levels *O*, *F*, and *M* based on the content of F, O, and M—all these are directly experienced in the normal state of mind. Say, we flash a picture of a guy called Dan for a 10 ms. The observer can easily report visual features just experienced or that an object was shown or that it was a human face. However, the report of the interpretive aspect based on the same features can go wrong—instead of reporting Dan the observer reports Chris with believing in being correct. As noted earlier, one of the most intriguing problems, but heretofore lacking solution, is this: how the already conscious sensory content in perception (step‐1 conscious access) becomes interpreted in conscious experience (step‐2 conscious access)? More specifically, where and how between the levels *F*, *O*, and *M*, the respective transform or modulation happens? Again, to reiterate, this stage‐wise processing, and especially its interpretive part is highly context‐sensitive.

#### 2.2.1 General Variety of Perceptual Processing Contexts

First of all, we want to distinguish whether contextual information is provided exclusively from the *internal mental sources* such as semantic or episodic memory, perceptual set, priming, tuning of selective attention, *or also from the actual sensory* information present alongside the object of interpretation. With regard to the canonical case of single (isolated) object interpretation all context is internal, memory‐based. In case when an object F_O_ is presented together with S_F_ that is not part of F_O_ (i.e., the embedded object context) we have a more complex situation where externally driven bottom‐up processes interact with top‐down processes. These top‐down processes would be a mixture of (i) the top‐down part of processing of the *actual* bottom‐up input and (ii) the top‐down processing predispositions present for this situation already *before* the object is presented. Another distinction of types of context is conceptual. Based on *conceptual characteristics*, figural‐categorical and semantic‐categorical types of context can be distinguished. (Figural‐categorical measurements in the conceptual domain strongly interact with spatial context measurements in the physical domain. Similarly, qualitative measurements in the physical domain can interact with semantic‐categorical measurements in the conceptual domain. Indeed, color orange as F_O_ is a non‐random associate of fruit orange as M_O_.) Although there are many ways for classification of the types of context, the one adopted here by no means overlooks the more or less common understanding of the topic (e.g., Brascamp and Shevell 2021).

Conscious interpretation of S heavily depends on what kind of sensory layout the observer is presented with. This is the sensory context of an object. The **types of S context** with important implications for explaining the process of interpretation can be classified as follows:

1. *Type‐* ***A*** *context—*a distinctly visible target form is part of a distinctly visible scene as one of its objects.
2. *Type‐* ***B*** *context—*certain set of sensory features is organized in a way allowing more than one object‐interpretation; that is, the case of ambiguous figures.
3. *Type‐* ***C*** *context—*certain object can be imagined based on the substantially incomplete set of its typological cues (e.g., Mooney faces, pareidolias); in certain cases, this happens even if the features represent a complete set of features of some other objects (some pareidolias).
4. *Type‐* ***D*** *context—*an object and (some of) its features are perceived even though they are not really presented among the other, well‐visible objects (e.g., hallucinations and illusory contours).

Current research on neural correlates of context effects on conscious interpretation includes a number of findings related to different types of contexts. In this paper, we focus on type‐A and type‐B contexts. Let me briefly review some of these studies next in order to give a general empirical background for our discourse. This is pertinent because our L5p‐based conceptualization is essentially a cognitive neuroscience conceptualization, albeit at a more microscopic level compared to typical neural networks‐based approaches.

#### 2.2.2 Examples of Contextual Effects From Recent Research

Aided by MEG recording and classification methods, Yan et al. (2023) presented participants with features of objects enabling two different categorical perceptions of the same stimuli. In cue‐feature training, feature‐category context was conditioned to auditory cues for each of the unambiguous instances of the images and ambiguous variant before the main experimental condition. Multivariate classifiers were trained to extract brain response markers for each type of percept. Yan et al. (2023) found that the conditioned predictive internal context effectively biased ambiguous image interpretation. The category‐specific *feature* level (F_O_) had a more distinct biasing effect on subsequent categorization behavior (aka interpretation by brain) than the typical category contrast. Internal, temporally preceding cognitive context effects may also happen spontaneously. For example, Hesselmann et al. (2008) carried out an fMRI study of neural correlates of Rubin's ambiguous vase versus faces figure perception (this is our S context variety **B**). Prestimulus activity in the fusiform face area turned out to be higher before subjects interpreted the stimulus as faces. Prestimulus context‐related spontaneous brain activity interacted with post‐stimulus activity in steering conscious interpretation.

One common and important type of sensory environment S providing *F*‐level data for processing consists in intact scenes with many objects included; objects present in noisy conditions (sensory context type‐**A**). In an MEG study, degraded objects were presented alone or included in scene context (Leticevscaia et al. 2024). Subjects either attended the scene or just fixated the fixation cross. Multivariate classifiers were preliminarily trained to distinguish clearly visible animate versus inanimate objects. It was found that at 300 ms after stimulus onset the MEG classifiers distinguished degraded objects in scenes better compared to when these objects were presented alone. Interestingly, while attention and context both had a facilitating effect on object category decoding, these effects were mutually independent. It appears that the processes capable of aiding object interpretation are multiple, with some of them working “automatically”, independent of the current task. In terms of levels of processing, it is not easy to precisely spot the level(s) responsible for these effects. In principle, there may be different explanatory processing modes: (i) facilitatory lateral interactions between *F*‐level features pertaining to background and target objects (F_backgr_ → F_O1_); (ii) similar lateral interactions, but between higher‐level representations (O_backgr_ → O_1_); (iii) top‐down facilitatory effects (O_backgr_ → F_O1_), possibly combined with (ii); (iv and iv+) different combinations of the top‐down/lateral effects of the conceptual level *M*.

Animal models are also used for research on brain mechanisms of context sensitivity. For example, stimulus novelty processing is regulated by internal context of learned associations and event probabilities. Hamm et al. (2021) recorded responses to “oddball” stimuli in the primary visual cortex of awake mice. Responses to redundant stimuli were reduced whereas deviant stimuli caused augmented responding. Importantly, and consistent with a cellular‐psychology approach emphasizing top‐down contextual modulation, optogenetic suppression of prefrontal inputs to V1 reduced the contextual selectivity of F‐tuned occipital neurons.

It is well known that basic level *F* attributes are encoded in early cortical areas (e.g., occipital cortex). Elegant experiments on contextual effects on early‐level conscious vision were conducted also by Rossel et al. (2022). The authors placed pictures of target objects either (i) in visual scenes that were contextually typical for the object or (ii) in the contexts prepared by transforming the images of (i) so that the object content of the scene was uninterpretable anymore, but the early‐level physical attributes were kept similar despite of the scrambling. (This is a variation of type‐**A** context.) The target object image was blurred at different levels. Observers’ task was to adjust the blur level of a comparison image of the target object so that the object in context and out of context would appear visually similar. The main result indicated that in order to look similar in terms of image sharpness, the blur level of the target object in the authentic scene context was adjusted to the level of higher sharpness than the target in the scrambled context. As edge sharpness is a property of images encoded early in cortex, contextual modulation of the visual appearance of O_x_ must have taken place also at the early level *F* of cortical F_x_ representations. One could ask why would the visual system want to modulate the early‐level representations, when it already has interpreted that object? What can possibly be gained here? Perhaps even if there is no behavioral gain, the system works this way by a default commitment: top‐down contextual modulation processes activate more when an otherwise well‐interpretable object is present alongside a multitude of other well‐interpretable objects in the scene. Perhaps lateral inhibition or higher‐level competition is stronger between objects than between an object and the surrounding objectless noise. This means that the target object has to be modulated in order to be more distinct.

Rossel et al. (2023) continued the same line of research with new results. Instead of letter strings, analog format pictures of objects were presented in the Lupyan's experimental paradigm. Additionally, the validity of expectations about objects was manipulated by varying the scene context. Valid expectations caused apparent sharpening of target objects when visual input was not reliable whereas with reliable visual input unexpected objects were perceived more sharply. Type‐**A** simultaneous valid context at the level of *O_x_* leads to improved *F*‐level mediated experience of the expected objects in more difficult perceptual conditions (type‐**A** with presentation in noise), but this changes to the advantage of unexpected object perception in less noisy conditions.

Conditioned visual hallucinations have been investigated in a couple of studies with different experimental settings (Aru and Bachmann 2017; Aru et al. 2018; Tulver et al. 2019; Vetik et al. 2020). In the initial stage of the experiments, many trials were presented where target objects (e.g., four rounded shapes with an inner area filled with two different colors, or female and male faces) were accompanied by task‐irrelevant objects (e.g., two rows of three small letters or a square‐shaped object not overlapping with targets). This is a type‐**A** contextual situation. The main task requested target discrimination, for example, whether two internal colored half were positioned similarly in all rounded shapes or was there an “oddball”, whether a female or male face was presented, or whether the faces had both the same orientation or not. In later trials, the task‐irrelevant objects were occasionally not presented, but subjects were, unexpectedly for them, asked to report whether they saw something else besides targets and also rate the subjective clarity of the odd stimulus objects on a 4‐point scale. It was found that in such trials subjects often hallucinated the presence of the actually absent odd object. Importantly, in quite many odd trials hallucinations were rated to have subjective vividness higher than the weakest clarity level. In terms of our notation, earlier conditioning of association between target object O_T_ and extra object O_E_ caused later hallucination of O_E_ when only O_T_ was presented. We cannot say for sure whether the mechanism of hallucination works exclusively at the *F*‐level by lateral (F_O1_ → F_O2_) facilitative interaction, at the stage of (O_1_ → O_2_) association with additional top‐down activation of F_O2_ from O_2_ above, or in some more complex mode (e.g., top‐down activation [O_1_ → F_O2_] combined with [O_2_ → F_O2_]). Preliminary results from brain imaging studies of hallucinations do not allow for conclusive theoretical clarity as yet (Collerton et al. 2023).

To preview the cellular‐psychology–based approach to the interpretation effects that will be presented later, it is worthwhile to remind the reader that context‐sensitivity is a core functional property of the L5p multicompartment neurons. According to the dendritic integration theory and apical amplification conceptualization (Aru et al. 2020a; Bachmann et al. 2020; Phillips 2017, 2023; Marvan et al. 2021), a sufficiently strong apical input originating from memory representations could produce cellular output by itself even in the absence of the feedforward sensory information. Hallucinations of O_F(S)_ are a model case of sensory experience without a corresponding input from level *S*. Indirect empirical support for the top‐down contextual input as the source of volatile perceptions comes from the method of direct artificial stimulation of the apical integration zone in awake mice (Takahashi et al. 2020, 2016). Applying optogenetic apical stimulation to L5p sensory neurons produced false alarm responses as if the animals were hallucinating the presence of an absent external stimulus.

There is an impressive number of studies with results sticking to the brain‐wise top‐down innervation theory of multistable, volatile interpretive account of context‐sensitive object perception (Bergmann et al. 2024; Murray and Herrmann 2013; Pang et al. 2021; Seghier and Vuilleumier 2006; von Gal et al. 2023).

#### 2.2.3 Examples of Contextual Disambiguation

**FIGURE 2:**
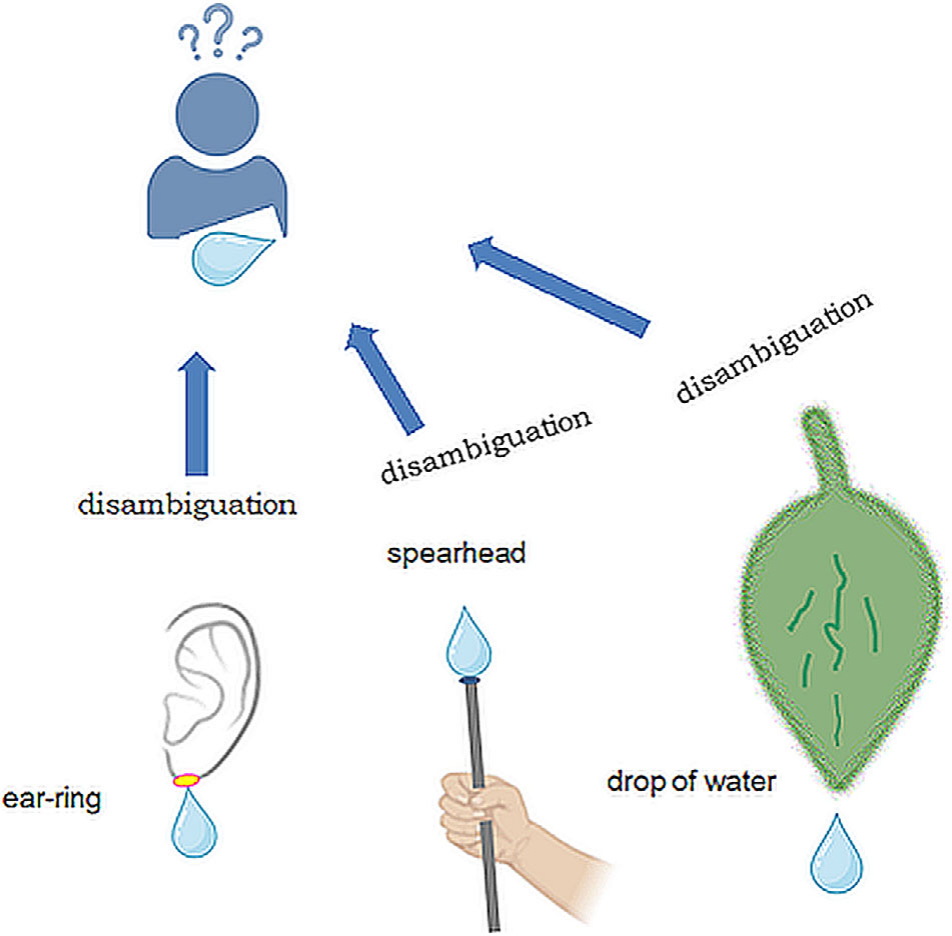
Examples of how the invariantly shaped object consisting of the same features F offers different interpretations to the observer depending on how this object is included in the scene for which it is a part.

Much more sensory input is ambiguous than we may expect. An important function of perceptual context is to help disambiguate ambiguous interpretations suggested by F_x_ and O_x_. Some cartoon‐level illustrations can be seen in Figure 2, and illustrative text in *Ambiguous image interpretation reversals*. However, it is not well understood whether context‐dependent disambiguation requires consciousness. Biderman and associates (2020) set to answer this question. The famous Bruner and Minturn stimulus design 13—“is it B or 13?”—was used so that the context providing flanking information was masked. Although effectively weaker, the context did bias perceptual interpretation of symbol objects also when presented at the unconscious level. However, lexical level context, set by words versus nonwords, required conscious level presentation in order to have an effect on target interpretation. In our present annotation, this means that effective disambiguation demands for context to be either at level *O* or lower “strata” of level *M*. Gori and colleagues (Gori et al. 2024) investigated contextual disambiguation of well‐known ambiguous figures—Rubin's vase/faces and wife/mother‐in‐law. However, the disambiguating context was presented by sound in the form of audible dialogue (for vase/faces target) or a young women speaking (for wife/mother‐in‐law). Despite the difference in modalities, the disambiguating effect of context on conscious interpretation was significant. Faces were more often perceived when primed by conversation than audible context without implicating the presence of people (a control condition). Wife was perceived more often than older woman when primed by a young woman speaking. The results are important for our purposes because they show that level‐*F* features need not be directly activated as the contextual priming source. In Gori et al. (2024), listening to people speaking must have activated level *O* visual representations of faces by input coming from the auditory *O*‐level representations, directly from auditory *M*‐level representations, or from visual *M*‐level representations activated by auditory representations (either from auditory *O*‐level or auditory *M*‐level).

The preceding paragraphs showed the diversity of empirical contextual interpretation effects. This diversity includes several varieties of *sensoriae* where object interpretation takes place. This happens by the involvement of level‐specific processing that can be comfortably related to the multicompartment L5p model of context‐sensitive processing as the apical compartment of L5p is specifically a context‐sensitive locus of modulation effects. Before coming to the description of how this model could explain context‐sensitive interpretation (so that at the same time the veridical F‐content will not be lost). I will list the classes of sensoriae for which this explanation is going to be used.

#### 2.2.4 Canonical Unambiguous Object Interpretation

In the canonical case of S we have an instance of a separate set of features joined (organized) into an entity commonly known as a definite kind of object (certain “thing”) and in this case the object is free from the actually present context of other objects and/or scattered features. The stage‐wise process unfolding in levels *S‐F‐O‐M* and representing content S‐F‐O‐M in this case culminates in an unambiguously interpreted object O_1_ associated with its meaning M_1_. For example, features “I” and “–” as put together in a specific way and present without other features in the visual field form an object “+” and become understood as “cross”.

#### 2.2.5 Interpretation of Objects as Parts of a Scene

In this case, type‐A sensoriae, features of object O_x_ are included in a scene. If the scene is unambiguously typical for object O_1_, the processing for interpretation is similar to what was described in the canonical case and culminates in unambiguous M_1_. However, if the scene is unambiguously typical for an object O_2_, even though the set of features forming this object as such is the same as for O_1_, the processing for interpretation culminates in unambiguous M_2_ (M_2_–M_1_). In Figure 2, three different cases of context are shown for illustration of how the immediate context biases the observer to have a specific visual‐cognitive interpretation of the featurally invariant depicted object, whereas other contexts suggest other specific interpretations despite F‐level object invariance.

(Probably there are exceptions where low‐level representations can withstand context effects on interpretation, but this does not overthrow the fact that many low‐level entities are multiple‐interpretable.)

#### 2.2.6 Ambiguous Image Interpretation Reversals

In this case, the features of some object O_x_ are ambiguous—the same set of features F can be used in the processing system for forming more than one type of object belonging to different visual and cognitive categories. Many well‐known examples include Rubin's vase/faces, Bugelsky and Alampay's rat/man, and the young/old woman figure widely known after Edwin Boring introduced it. (This kind of ambiguous object can be disambiguated by immediate context as was shown, for example, in the widely quoted paper by Bruner and Minturn (1955). The symbolic object 13 is perceived as a letter B in the sequence A 13 C and as a number 13 in the sequence 12 13 14.) The possible processing options for interpretation after level *F* bifurcate: content F_x_ becomes interpreted either as O_1_ or as O_2_. Even though the set of features forming the proto‐object O_x_ is the same for O_1_ and O_2_, there has (have) to be some process(es) that make(s) it possible to select only one interpretation at a time. This could occur in several principled ways. First, by modulating O_x_ so that the visual sensory‐phenomenal emphasis in some way helps selectively perceive one of the alternative interpretations (e.g., O_2_) realized at level *O*. This could be carried out by additional selective facilitation of the O_1_ representation without sufficient activity of the O_2_ representation (activating and later feeding also the corresponding M_1_). Second, the processing of the feature set of O_x_ at the sensory, phenomenal, and visual Gestalt object level remains invariant, but alternative interpretations become realized at level *M* either as unambiguous M_1_ or unambiguous M_2_.

**FIGURE 3:**
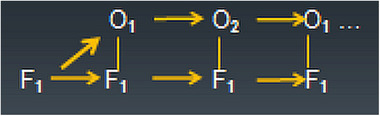
Schematic flow‐chart for information processing at levels F and O where interactive activation of invariant F_1_ representations and alternative O_1_ and O_2_ representations takes place. The same set of features F_1_ allows for alternative interpretations O_1_ (real object) and O_2_ (pareidolic object). (Arrows mark progression of time epochs.).

Schematically, in the present case of type‐**C** of the ambiguous input variety of S the interaction of level‐*F* and level‐*O* processes in allowing the perceiver to interpret a given set of features F_1_ according to the first of the above‐processing versions would seem to look like as symbolized in Figure 3. Throughout the whole episode of phenomenally experiencing the features F of an ambiguous object O_amb(x)_ all features are invariantly *present* in perception. However, some process of putting selective *emphasis* on selected features pertaining to one interpretation (e.g., O_1_ vs. O_2_) does the job for disambiguation and invariant extraction of one of the objects. Because of this possibility we would be enforced to modify the notation F_1_ in our schematic shown in Figure 3 so that F_1a_ and F_1b_ would correspond to the two competing versions of selective feature‐*emphasis* (i.e., without abandoning the features themselves), respectively, for O_1_ versus O_2_ content. This allows not only the switch from one to the other interpretation, but also the typical reversal‐prone alternation of conscious interpretations observed in virtually all known ambiguous stimulus images. (Whether this is accomplished just by selective Gestalt grouping delineating the set of one object among the two potentially possible objects remains an open question. Some sort of selective modulation of subjective vividness of a selected set of features remains a possibility consistent with the core mechanisms adopted currently in cellular psychophysiology. We will return to this issue in a more detail later on.)

In the above‐listed kinds of *sensoriae* (S) from type‐A and type‐B, context plays a highly important if not decisive role in steering object O_F_ interpretations.

Now, after having reviewed a range of types of context, studies of context effects and principle processing flow‐charts across processing levels and representations let us turn to the question: how cellular psychology as focused on L5p multicompartment context‐sensitive neurons could explain various contextual interpretation effects.

## 3 Results

### 3.1 Explaining Conscious Interpretation by the L5p Contextual Modulation Mechanism

**FIGURE 4:**
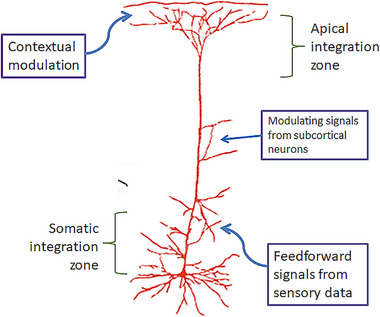
Layer‐5 pyramidal multicompartment neuron (L5p) with thick‐tufted apical dendrites in the cortical layer 1 and its main sources of presynaptic input. The combined input to the dendrites in the perisomatic zone (in layer 5), apical zone (in layer 1), and intermediate coupling zone (receiving signals from subcortex) together with precise temporal coordination of arrival of the inputs from these sources controls postsynaptic potentials and firing pattern of the L5p. Apical and perisomatic zones can respond autonomously to their inputs. However, their coordinated action in response to bottom‐up signals, contextual modulation signals, and signals from subcortex (controlling to what extent perisomatic and apical zones’ actions can be integrated) determines how much the perceptual content for which the cell is specialized becomes accessed at the conscious level.

For the cellular psychology‐driven explanations of different types of context effects let me use a simplified version of the multicompartment L5p neuron model (Figure 4). Let us not forget that cellular psychology does not adhere to the traditional single‐unit, cardinal cell type of theories regarding single‐cell highest‐level units as the true representations for specific percepts (review: Quiroga 2013). It is assumed here that the single‐cell model together with its immediate presynaptic neurobiological microenvironment is meant to be used for showing how a relatively large population of such neurons with common function provides a mechanistic explanation for mental events. In what follows we will present hypothetical mechanistic explanations for object interpretation in different types of sensory context as based on the multicompartment L5p neuronal model.

#### 3.1.1 Type‐**A** Sensory Context

In this case, the object of interpretation (O_int_) is part of a scene of certain other well‐visible objects that together form a general external context of interpretation. The level of ambiguity for this object is very low because of the natural consistency between context‐forming objects and the to‐be‐interpreted object (illustration: Figure 2). The prevailing majority of contextual signals to the apical compartment of the dendrites of L5p(O_int_) (i.e., the L5p tuned to O_int_) originating from representations of the level *M‐* and level *O* representations of the context‐objects cause apical zone spikes in L5p(O_int_). Depending on what are the context objects, the distant sources of apical modulation of L5p(O_int_) are different and specify any particular context, which associates the activity of the set of the “ambiguous” neurons L5p(O_int_) specifically with the set of neurons L5p(O_x_) that represent the context objects. The basal, perisomatic compartment of L5p(O_int_) at the same time keeps receiving sensory input from level *F* of O_int_. There is a high‐level learned consistency between the represented perceptual contents of the context‐carrying neurons (that stimulate apical dendrites of L5p(O_int_)) and the specific attributes of the object represented by L5p(O_int_) by the activity of its basal dendrites. Therefore, the burst spiking of L5p(O_int_) is strong and keeps the information about object O_int_ integrated within the currently perceived scene as its natural part. Should the set of context‐objects change while the principled consistency between context and O_int_ features is not violated, then the same set of features of O_int_ can be validly, albeit differently, interpreted at level *O*. However, let us keep in mind that in this case the sources of apical modulation of L5p(O_int_)) are different from what they were with different object interpretation. Feature‐neurons remain the same, but the sources at the higher object level from where the top‐down contextual input converges on the feature neurons are variable. Therefore, the apparently same sensory identity for the object features at level *F* will be differently interpreted at level *O* as a valid part of the *different* context. In terms of neurobiological cellular mechanisms the selection of context that modulates the apical L5p compartment is likely to be mediated by a special type of interneurons (e.g., Almeida 2022, pt 3.4.1–3.5; Marvan and Phillips 2024; Phillips 2023). These inhibitory interneurons (SST/SOM, VIP, NGF) receive input from top‐down and lateral sources and, in turn, send modulating efferents to the L5p apical compartment. Depending on the specific *O*‐level or *M*‐level source of presynapting effects on interneurons, the “ambiguous” L5p bursting will be integrated with activity of the neurons representing the scene context. (Let us bear in mind that if inhibitory interneurons are inhibited, the spiking activity is enhanced, leading to L5p activation.)

#### 3.1.2 Type‐**B** Sensory Context

In the case of the multiply interpretable (ambiguous) visual structures, the *F*‐level set of L5p neurons tuned to features of the to‐be‐interpreted object O_int_ is invariant. The invariant feedforward inflow of signals from *S* is directed at the perisomatic zone of L5p(F(O_int_)). However, despite this feature‐invariance, the direction of selective activation of object representations at level‐*O* has more than one option (unlike the canonical situation with one unambiguous object presented in isolation). The set of higher‐level L5p(O_int_) neurons includes different subsets, each tuned to its own object‐interpretation O_x_, for example, O_1_ and O_2_. The multistable interpretation activity is hypothetically realized as follows. When object O_1_ is dominating in conscious perception, it means that level‐*F* neurons L5p(F(O_1_)) have received additional apical zone activity amplification via top‐down pathways from O_1_ representing units (e.g., L5p(O_1_)) which results in relatively higher subjective emphasis of the subset of L5p(F(O_1_) in conscious perception. (Presumably the sources of L5p(F(O_1_) apical modulation have provided an additional effect of amplification via VIP → SST interneurons applied at the apical calcium spike zone and apical tuft. As a result, the relative level of burst activity has become selectively increased for the set of L5p(F(O_1_) that represent the features of the object O_1_ currently dominating in awareness.) Let us keep in mind that features of O_2_ (represented by L5p(F(O_2_)) keep being consciously represented in parallel with O_1_ perception; however, as supported by a relatively lower level of apical‐zone activation, the latter being carried out by background level of presynaptic top‐down influence. As soon as the net apical‐zone activity of L5p(F(O_2_) becomes higher than that for L5p(F(O_1_), the selective vividness enhancement for O_2_ causes it to be interpreted as the content of current *S*. This switch from perceiving O_2_ instead of O_1_ from data presented by an invariant *S* and invariant *F*‐level input signals may have different reasons—such as inhibition‐causing adaptation effects on L5p(F(O_1_), disamplification of L5p(F(O_1_) activity, disinhibition of L5p(F(O_2_), top‐down selective facilitation effects from level‐*M* on L5p(O_2_), and this list of possibilities should not be considered to be exhaustive. (See Phillips 2023, on the varied L5p‐related micromechanisms carrying out the contextual modulation effects, including both facilitatory and inhibitory ones.) Future research should be directed at disentangling these hypothetical mechanisms and exploring their mutual cooperation and competition regimens. Switches between interpretations are all‐or‐none and subjective vividness difference between O_1_ and O_2_ features is gradual (non‐dominant object features are not fully “blinded”, but relatively less vivid). This suggests that the EEG‐markers for an ambiguous image to become phenomenally conscious (NCCthr) could show graduality (exemplified by visual awareness negativity (VAN), perception awareness negativity (PAN), or N170 amplitudes) whereas the NCC for interpretation switch could be found in the ERP components sensitive to categorical decisions.

How could bistability of ambiguous figures as an instance of categorical decisions be explained by the neurobiological L5p modulation? I am grateful to one anonymous reviewer who suggested a specific example of such image and recommended explanation. Let us take Rubin's vase versus faces image. If attention is focused on the central part of that figure, a vase representation may be amplified; a less focused gaze could promote greater contextual integration of surrounding sensory signals for visualization of the faces instead. How apical modulation mechanisms could explain this psychological effect? As noted earlier, the L5p dendrite can be modulated by inhibitory interneurons such as somatostatin‐positive SST. Surround suppression via SST is thought to promote an averaging of pyramidal cell responses, promoting a Gestalt‐level perception by way of divisive normalization (Shin and Adesnik 2024). On the other hand, the type‐VIP inhibitory interneurons focalize smaller stimuli in order to amplify them at the expense of divisive normalization (e.g., Millman et al. 2020). Hypothetically, competitive interaction of VIP and SST modulation could drive bistability: by shifting attentional focus to the center of vase area we might amplify the vase representation, whereas by more peripheral attention we could promote greater contextual integration of surrounding information for coming to interpret the image as faces instead. This example reminds again that the cellular‐psychology L5p model is not meant to ascribe consciousness to one cell, but shows how single cells can function as crucial elements in the population‐level processes critical for conscious experience. Net apical amplification in a selective set of many L5p‐s is what makes a coherent Gestalt.

The hypothetical explanations for perceptual interpretation multistability in the face of continuous conscious‐level presence of *F*‐level features of both alternatives need not be restricted to selective apical modulation exclusively at this level. As we are far from knowing the cellular level NCC precisely enough, other possibilities are there to be investigated. For example, even without the *F*‐level selective apical amplification, there are other mechanistic explanations of conscious interpretation possible. If the NCC that are sufficient for conscious perception include combined (“transparently overlayed”) activity of *F*‐level and *O*‐level representations then it is possible that selective emphasis, while lacking at the *F*‐level L5p is realized at level *O* only. All features F (both, for O_1_ and O_2_) are brought to conscious experience with equal magnitude of apical amplification at level *F*, but level *O* enjoys selective apical activation of different L5p(O_x_) alternately, that is, in turns for L5p(O_1_) and L5p(O_2_). Perhaps it would be an effect of some winner takes all type mechanism working autonomously at level *O*. This possibility is nicely consistent with the data on typical temporo‐occipital coordinated activity with prevailing recurrent interaction between levels when ambiguous images are perceived (Brascamp and Shevell 2021; Rassi et al. 2019; Wang et al. 2017). Whatever the precise mechanistic explanation of the resolution of ambiguity may be, the involvement of multilevel mechanisms in ambiguous figure perception is a generally accepted theoretical stance (Long and Toppino 2004).

### 3.2 Useful Properties of the L5p Model

In the preceding parts, we envisaged the specific mechanistic “algorithms” of L5p activity for context‐sensitive disambiguation and conscious interpretation. However, we believe there are also more general uses of the cellular psychology approach for the brain‐based science of consciousness. Although there are many influential theories of consciousness with their central focus on NCC (Storm et al. 2024), there has been no progress in clearly distinguishing mechanisms of NCCthr and NCCint (Bachmann and Aru 2023). Among the numerous issues accompanying the prevailing theories that probably make obstacles for the needed progress we can see some crucial ones. As the issues are listed, comments will be given in each item on what useful property the cellular‐psychology approach can add to the development of brain‐based consciousness theories. (1) The somewhat too selective empirical basis (e.g., certain experimental paradigms) brought to support each specific theory seriously compromises generalizability and carries an ad hoc flavor (Chis‐Ciure et al. 2024; Evers et al. 2024; Promet and Bachmann 2024; Storm et al. 2024; Yaron et al. 2022). The DIT and AA processing models could add a new level of experimental research suitable for more precise testing of the neural mechanisms involved in producing a number of consciousness phenomena (Bachmann 2015; Marvan et al. 2021; Phillips 2023); these theories could be resourceful for overcoming mutual inconsistency between different—mostly molar—consciousness theories by showing a mechanism that is likely common to all major theories (Storm et al. 2024). (2) By and large, different theories have had stronger focus and empirical foundation either on contents of consciousness (e.g., Crick, Lamme, Rosenthal, Dehaene, Seth, Hohwy) or consciousness states (e.g., Tononi, Massimini, Albantakis, Mashour, Northoff, Llinás). The AA mechanism and DIT (with double “echo” at the perceptual retouch theory—Bachmann 2021) explicitly explain how the L5p mechanism is involved both in activating consciousness contents and regulating the states of consciousness (Bachmann and Hudetz 2014; Phillips et al. 2024). (3) Consciousness is a general gnoseological kind and kind of reality that is pervasively universal and pan modal. Phenomenality covers all senses and it seems that most of the brain systems are involved in how the brain creates conscious experience. On the other hand, different brain systems are quite separated in space and different sensory‐perceptual areas are tuned to physically different sensory signals. The large‐scale inter‐areal connectivity‐communication theories of consciousness such as different global workspace versions have some difficulty in explaining how consciousness unity is dug out of this diversity. However, the facts that multicompartment neurons (i) populate virtually all cortical areas, (ii) are a central unit in interlinking between‐areas activity, (iii) are universal in terms of neurobiological functioning principles, (iv) are sensitive to all main neuromodulators known to manipulate states and content of the mind, and (v) are tightly under genetic control in terms of their development (and we know how striking the individual differences in norm and neuropsychiatric pathologies can be)—all this suggests that there is some good logic in ascribing a highly important role to the L5p mechanism for consciousness. (**4**) Virtually all consciousness theories accept that the mind functions in two main regimens—the domain of unconscious states and the domain of conscious states, with a two‐way transfer between them. The multicompartment build‐up nature and autonomy of activity of different compartments of L5p make it a natural solution for explaining how unconscious, preconscious and conscious states are different, but permanently interact. For example, neuroplasticity allowing information storage in the “unconscious state” of the neuron and the capacity of the neural cell for graded (including “unconscious”) changes in the neuroelectric potential as combined with the dendritic activity‐coupling principle is a believable solution to the various basic problems, for example, how do the unconscious and conscious processes interact. (5) Part of the consciousness theories tend to be more “top‐down minded”, especially stressing the frontal‐cortex control (e.g., Dehaene, Rosenthal). Others give more due to more caudal cortical areas (Lamme, Koch, Tononi). Moreover, some theories give only a very general enabling role to the subcortical arousal‐steering systems such as thalamus (HOT, global workspace) whereas other theories accept that subcortex plays a central role not only in general arousal, but also in mediating access to specific conscious content. The L5p‐centered DIT and AA models seem more balanced in this respect by attributing an equally important role to cortico‐cortical and cortico‐subcortical mechanisms and feedforward as well as top‐down varieties of processing. This is achieved by the fact that L5p is a hub integrating all these different mechanisms. (6) There are some unsolved problems of cognitive penetrability of sensory phenomenal experience and phenomenality of the access consciousness. As the conscious mind has access to all levels of detail and granularity of the perceptual phenomenal experience, but the access control function has been delegated to the frontal‐cortex mechanisms of cognitive control, there is a puzzle: how the frontal areas that—as we know so far—lack narrowly tuned neurons with very small receptive fields are capable of “knowing” the perceptual detail phenomenally and/or receive the “broadly broadcast” sensory content in case of the fine‐detailed stimulus content. (Some of these problems are nicely outlined by Block 2024, and Kozuch 2024.) The L5p‐centered theory permits a solution to this conundrum. The combined activity of the apical compartment (the carrier of the top‐down control signals’ effects) and perisomatic compartment (the carrier of the detailed sensory information) harmonizes conscious access and fine‐scale direct phenomenal representation. Relatedly, the autonomy of attention and awareness mechanisms combined with their capacity to interact also surrenders to scrutiny by AA. Preconscious attentional effects can take place autonomously at the apical compartment of L5p without necessarily leading to current rigorous spiking. Conversely, the attention invariant phenomenal level of expression of perception (e.g., “vividness”) may be mediated by the autonomous change of the level of activity in the perisomatic cell compartment without differences in the top‐down attentional involvement by the apical modulation. (7) Recent research has shown that many serious neuropsychiatric conditions are associated with anomalous expression of the conscious mind as well as with pathologies in the neuromodulation where L5p is visibly implicated (Almeida 2024; Granato et al. 2024; Phillips 2023). Unfortunately, we do not have space for delving into this domain here. Suffice it to say that research in cellular‐level mechanisms as NCC must be informative also for the health‐related applied domains.

The useful properties of AA and DIT as well as cellular psychology in general are noted also in Storm et al. (2024), Phillips (2023), and Phillips et al. (2024). For example, the L5p neurons do associate information from apical and basal compartments by non‐linear interaction rules, which are dependent on dendritic voltage‐sensitive ion channels. Importantly, this kind of apically directed control is executed by highly specific targeted inhibition and neuromodulation. This allows highly differentiated content forms of conscious experience. Furthermore, according to DIT, prior knowledge (our levels *O* and *M*) relates to the associations with perceptual processes stressing the unity of contentful memory and perception via the same structural microlevel units. Influence of synapses linking apical compartment to the rest of the system fosters semantic memories as stored in the layer‐1 connections (Shin et al. 2021).

Most importantly, as seen from the aim of this paper, the autonomy of apical and other L5p membrane compartments appears as a natural solution to the phenomenal fact that *invariant* sensory content of the percept can coexist with the *varying* interpreted content of the stimulus object.

### 3.3 Possible Directions of Research for Testing the Cellular‐Psychological Explanation of Conscious Interpretation

As we just noted, the main puzzle in trying to find specific mechanisms of NCCint as distinct from NCC for *F*‐level content consists in the coexistence of *F*‐level content *invariance* with *O*‐level (and also *M*‐level) content variance (Bachmann and Aru 2023). In the preceding parts of this paper, we saw that the cellular‐level L5p multicompartment model of context‐sensitive processing suggests a viable hypothetical mechanistic explanation. The capacity of autonomous functioning of basal (sensory data encoding) and apical (context‐sensitive) dendritic zones allow certain independence and non‐linear interaction of *F*‐level information processing and higher‐level information processing. Consequently, in order to experimentally test the hypothetical L5p multicompartment model of conscious interpretation, independent or precisely combined experimental manipulation or activity recording of the state of two integration zones can be used.

There are several promising methods to be used for the realization of the above‐mentioned research strategy. For example, we can take advantage of the known endophenotypal differences in the relative expression of micromechanisms that participate in the control of layer‐1 dendritic modulation and layer‐5 perisomatic modulation. It is known that noradrenergic modulation, which is related to NMDA receptor‐mediated process, is strongly involved in establishing behavioral priorities, that is, in selectivity of responses (Mather et al. 2016; Shine 2023). Apical amplification is a mechanism effectively supporting this process (Larkum and Phillips 2016). Predictably, if apical dendrite spiking of L5p tuned to O_2_ (and not O_1_) will be stimulated by optogenetic techniques or electrophysiological methods in the conditions where (Σ_F(O1)_ = Σ_F(O2)_) as encoded by the simultaneous bottom‐up signals inflow to basal dendrites, conscious interpretation of O_x_ should either switch from O_1_ to O_2_ or stay with O_2_. The individual differences approach can also be used. If the behavioral dynamics of perceptual interpretation would differ between subjects prone to strongly expressed apical modulation and subjects with relatively weak expression of this endophenotype, the present theory would get additional support.

Cellular membrane processes of L5p are controlled by a number of different neuromodulators (e.g., Shine 2023). Facilitatory neuromodulation of superficial layers of neocortex is a well‐established capacity for such agents as mACh, aminergic neuromodulators (NA/NE, DA, 5‐HT), and the glutamatergic mGluR. Experimental manipulation of neuromodulation in experiments with conscious interpretation can shed more light on NCCint mechanisms. Gelbard‐Sagiv et al. (2018) conducted a study with S‐context‐free placebo‐controlled experiment on visual perithreshold target perception where activity of the adrenergic system was manipulated pharmacologically. Behavioral and psychophysiological results demonstrated sensitivity increase and NCC neuromarker augmentation. It would be nice to use this method also with in‐context targets and ambiguous image disambiguation by context‐driven interpretation.

By almost common knowledge, autistic traits are associated with difficulties of disengagement from sensory‐specific perceptual cues and perception of ambiguous sensory stimuli; this is characteristic of individuals for whom serotonergic system functioning is somewhat compromised (Vollenweider and Preller 2020; Zimmerman et al. 2008). Some of the autism spectrum disorders cause difficulties in contextual interpretation and context switching. Future research could shed light on whether pathology or functional inefficiency of the L5p multicompartment dendritic interaction mechanisms might be involved here. (Actually, in one of their experiments, Aru et al. (2018), studied “normal” hallucinations of subjects who also completed the Autism Spectrum Quotient questionnaire. There was a negative correlation between the amount of hallucinations and the test score—subjects with higher scores were less likely to experience illusory objects. We can speculate that these subjects were less disposed to have apical drive caused by earlier associative context.)

Individual neurobiological endophenotypes and behavioral phenotypes of expression of neuromodulation are in turn influenced by genetic variability (e.g., individual SNP variants). It is logical to expect that this kind of individual variability also predicts variability in the dynamics of conscious interpretation of multiply interpretable S if apical amplification in L5p is strongly involved. As noted earlier, the 5‐HT system is one of the major neuromodulation systems in the brain. It is involved in apical amplification because this system is strongly expressed in the apical layers of cortical layer‐5 pyramidal cells, includes 5‐HT receptors that induce excitatory postsynaptic potentials in apical dendrites and it also causes cortico‐thalamic activation of arousal system‐related “non‐specific” nuclei in subcortical loci (Aghajanian and Marek 1997; Marek and Schoepp 2021; Shine 2023; Vollenweider and Preller 2020). Therefore, experiments on conscious context sensitive interpretation with independent variables associated with serotonergic brain systems should be advisable to advance cellular theories of conscious interpretation.

Metacontrast masking studies with groups of subjects genotyped for variability in different serotonergic system‐related SNPs have been recently conducted (Maksimov et al. 2013, 2015; Maksimov et al. 2017). Masking provides a specific temporal context of fast‐alternating differently shaped stimuli with a peculiar kind of ambiguity—two different objects occupy virtually the same space within a time interval too short for realistic exchange of different objects there. This is ontologically an impossible situation and the human brain has to somehow solve this impasse in order to provide the perceiver with some cognitive solution. Typically in masking, the subsequent shape‐object wins and the preceding one is lost from conscious perception (provided optimal visual contrast and onset asynchrony) (Bachmann and Francis 2014). Maksimov et al. (2013, 2015) and Maksimov et al. (2017) showed that qualitative and quantitative differences in masking functions depended on variants of serotonin expression‐related SNPs. Human or animal masking experiments with direct recording/control of the level of involvement of L5p dendritic mechanisms must shed light on whether these differences may have been indeed caused by individual differences in the spiking patterns of multicompartment cells. (Interestingly, some difference in the results was caused by whether the target and mask global shapes were mutually congruent or incongruent. This could hint at a certain similarity between normal population masking mechanisms’ sensitivity to serotonergic neuromodulation on the one hand and the involvement of serotonergic neuromodulation in the context disambiguation processes specific to autistic traits on the other hand. Why so? Autistic predisposition is known to be intolerant to sensory ambiguity. In both cases the causally involved mechanism is quite likely associated with neuromodulation targeted at the apical zone of the dendrites.)

Of course, the serotonergic neuromodulation mechanisms in L5p are much more complex than the simplified and hypothetical considerations as drawn above (review: Marek and Schoepp 2021). One has to take into account (and experimentally control in future studies) the related glutamatergic neuromodulation's share in apical amplification, counteracting non‐5‐HT_2A_ type receptor effects, and so forth. Nevertheless, there are already more specific descriptions of how the cellular‐level micromechanisms and different types of neuromodulation mediate apical amplification and dendritic integration in L5p (e.g., Phillips 2023; Shine et al. 2021; Whyte et al. 2024). A big part of this argument is hypothetical, but the quite specific targets for future experimental stimulation/recording studies are clearly outlined. (Some future directions are pointed out also in Phillips et al. 2024).

In this theoretical review, virtually all examples and cellular‐psychology model applications have been unimodal, on vision. It should be urgent to treat analogous issues also in audition and touch, for example. Moreover, motivated by one reviewer we can ask whether phenomena of intermodally presented stimulation interpretation sustain the rule, “interpretation changes while sensory‐level content remains unchanged” as applied for ambiguous stimuli? For example, in one of the McGurk effect (McGurk and MacDonald 1976), demonstrations auditory interpretation of a heard stimulus (e.g., ‘ba‐ba’) becomes erroneously perceived (e.g., heard as ‘da‐da’) when visible mouth of the speaker utters a different syllabic content (e.g., ‘ga‐ga’). By this change of interpretation, correct auditory sensory information becomes lost and replaced by an illusory content. The visual interpretation causes change in *F*‐level auditory phenomenal content. Perhaps then it is time to question whether the continued presence of lower‐level feature content with changed higher‐level interpretation holds also for non‐visual modalities and intermodal stimulations with ambiguity being present. Remarkably, the McGurk effect has been found also when the mouth region of the pixelized speaker face is depicted only by 6–8 pixels (MacDonald et al. 2000). This means that, for example, the non‐face like 3 × 2 pixels had to be interpreted as a mouth in the context of pixelized talker face in the condition where *F*‐level features are inconsistent with *O*‐level representation. This in turn hints at the possibility that for the “simultaneous *F*‐ and *O*‐level representations’ experience”, rule, to hold these representations must include mutually consistent learned content.

The author cannot escape one more commentary on how the L5p multicompartment context‐sensitive mechanism may be involved in conscious interpretation. It is well confirmed that frontal L5p are the principled source of corticothalamic pathways to subcortex, including several nuclei known to be intimately associated with the state‐ and content‐modulation for consciousness—such as the intralaminar/midline thalamic nuclei, basal ganglia, brainstem/pons nuclei, and so forth (Marek and Schoepp 2021; Shine et al. 2023). As indicated in these and many other reviews, it is also known that activation of the cortex by general arousal‐servicing neuromodulation takes place over wide areas in all lobes of the brain massively populated by L5p. Notably, sensory input evokes the arousal response from adrenergic and cholinergic subcortical sources with activation projected massively to frontal areas and then down to more caudal cortical zones. This means that in addition to top‐down contextual modulation signals (i) from the *specific content*‐related frontal areas *as directed to apical dendrites* of L5p at the more caudal cortical levels a substantial share (ii) of the top‐down influence can be just expression of general arousal which is non‐specific to the contents. It is important in future research to disentangle top‐down effects that are content‐specific already from the highest areas carrying the content information and top‐down effects that are the result of cortico‐cortical modulation, but as a general signal to all units that “are interested”. This would be a different type of non‐specific modulation in addition to what has been shown to take place when higher‐order thalamic nuclei regulate L5p activity by controlling facilitatory influence *directed at the intermediate dendritic zone* between somatic and apical compartments (Phillips 2023; Suzuki and Larkum 2020). All this is not trivial because even though the modulation comes from subcortical neurons that themselves are non‐specific to content, their effects at the cortical level can be highly specific content‐wise. If the contextual information impact is set simply by a “subliminal” effect (i.e., without currently occurring burst spiking) on the L5p by differences in the cell depolarization level (or concentration of neurotransmitters) for contextually valid versus invalid specific L5p then the context‐*specific* effect can also be achieved when the arousal‐mediated modulation originates from *non‐specific* sources.

## 4 Conclusions

Earlier research has had no success in explaining what is different in NCCs which stand for phenomenal experience of the sensory content (NCCthr) of an object compared to NCCs that would stand for conscious interpretation of this sensory content (NCCint). Additionally, as conscious interpretation is highly‐sensitive to the current context where the target object appears, the notions of context sensitivity and disambiguation were shown to be important to deal with when we want to discover the clear distinction(s) between NCCthr and NCCint. Supported by the objectively specified types of contexts and description of the multicompartment single‐neuron model of context sensitive processing by L5p we showed in this paper the hypothetical modes of processing explaining conscious interpretation. The ideas behind this paper and the explanatory mechanistic model are essentially the same as accepted and understood in the newly emerging field of inquiry—the cellular psycho(physio)logy (Almeida 2022; Aru et al. 2020a; Bergmann et al. 2024; Granato et al. 2024; Phillips 2023; Phillips et al. 2024). Hopefully, the framework and ideas presented here may be instrumental in the purposeful experimental research aimed at distinguishing NCCthr and NCCint in the studies to follow.

## Author Contributions

**Talis Bachmann**: Conceptualization; investigation; writing – original draft; methodology; validation; visualization; writing – review and editing.

## Conflicts of Interest

The author declares no conflicts of interest.

### Peer Review

The peer review history for this article is available at https://publons.com/publon/10.1002/brb3.70393.

## Data Availability

Data sharing is not applicable to this article as no datasets were generated or analyzed during the current study.

## References

[brb370393-bib-0001] Aghajanian, G. K. , and G. J. Marek . 1997. “Serotonin Induces Excitatory Postsynaptic Potentials in Apical Dendrites of Neocortical Pyramidal Cells.” Neuropharmacology 36, no. 4‐5: 589–599. 9225284 10.1016/s0028-3908(97)00051-8

[brb370393-bib-0002] Almeida, V. N. 2022. “The Neural Hierarchy of Consciousness: A Theoretical Model and Review on Neurophysiology and NCCs.” Neuropsychologia 169: 108202. 35271856 10.1016/j.neuropsychologia.2022.108202

[brb370393-bib-0003] Almeida, V. N. 2024. “Somatostatin and the Pathophysiology of Alzheimer's Disease.” Ageing Research Reviews 96: 102270. 38484981 10.1016/j.arr.2024.102270

[brb370393-bib-0004] Aru, J. , and T. Bachmann . 2017. “Expectation Creates Something Out of Nothing: The Role of Attention in Iconic Memory Reconsidered.” Consciousness and Cognition 53: 203–210. 28687418 10.1016/j.concog.2017.06.017

[brb370393-bib-0005] Aru, J. , T. Bachmann , W. Singer , and L. Melloni . 2012. “Distilling the Neural Correlates of Consciousness.” Neuroscience & Biobehavioral Reviews 36, no. 2: 737–746. 22192881 10.1016/j.neubiorev.2011.12.003

[brb370393-bib-0006] Aru, J. , M. Suzuki , and M. E. Larkum . 2020a. “Cellular Mechanisms of Conscious Processing.” Trends in Cognitive Sciences 24, no. 10: 814–825. 32855048 10.1016/j.tics.2020.07.006

[brb370393-bib-0007] Aru, J. , F. Siclari , W. A. Phillips , and J. F. Storm . 2020b. “Apical Drive—A Cellular Mechanism of Dreaming?.” Neuroscience & Biobehavioral Reviews 119: 440–455. 33002561 10.1016/j.neubiorev.2020.09.018

[brb370393-bib-0008] Aru, J. , K. Tulver , and T. Bachmann . 2018. “It's All in Your Head: Expectations Create Illusory Perception in a Dual‐Task Setup.” Consciousness and Cognition 65: 197–208. 30212753 10.1016/j.concog.2018.09.001

[brb370393-bib-0119] Aru, J. , M. Suzuki , R. Rutiku , M. E. Larkum , and T. Bachmann . 2019. “Coupling the state and contents of consciousness.” Frontiers in Systems Neuroscience 13: 43. 31543762 10.3389/fnsys.2019.00043 PMC6729974

[brb370393-bib-0010] Bachmann, T. 2015. “How a (sub) Cellular Coincidence Detection Mechanism Featuring Layer‐5 Pyramidal Cells May Help Produce Various Visual Phenomena.” Frontiers in Psychology 6: 1947. 26733926 10.3389/fpsyg.2015.01947 PMC4686615

[brb370393-bib-0011] Bachmann, T. 2021. “Representational ‘Touch’ and Modulatory ‘Retouch’—Two Necessary Neurobiological Processes in Thalamocortical Interaction for Conscious Experience.” Neuroscience of Consciousness 2021, no. 2: niab045. 34925911 10.1093/nc/niab045 PMC8672242

[brb370393-bib-0012] Bachmann, T. , and G. Francis . 2014. Visual Masking: Studying Perception, Attention, and Consciousness. Elsevier/Academic Press.

[brb370393-bib-0013] Bachmann, T. , and A. G. Hudetz . 2014. “It Is Time to Combine the Two Main Traditions in the Research on the Neural Correlates of Consciousness: C = L× D.” Frontiers in Psychology 5: 940. 25202297 10.3389/fpsyg.2014.00940 PMC4141455

[brb370393-bib-0014] Bachmann, T. , M. Suzuki , and J. Aru . 2020. “Dendritic Integration Theory: A Thalamo‐Cortical Theory of state and Content of Consciousness.” Philosophy and the Mind Sciences 1, no. II. 1–24.

[brb370393-bib-0015] Bachmann, T. , and J. Aru . 2023. “Conscious Interpretation: A Distinct Aspect for the Neural Markers of the Contents of Consciousness.” Consciousness and Cognition 108: 103471. 36736210 10.1016/j.concog.2023.103471

[brb370393-bib-0016] Bar, M. 2021. “From Objects to Unified Minds.” Current Directions in Psychological Science 30, no. 2: 129–137.

[brb370393-bib-0017] Bergmann, J. , L. S. Petro , C. Abbatecola , M. S. Li , A. T. Morgan , and L. Muckli . 2024. “Cortical Depth Profiles in Primary Visual Cortex for Illusory and Imaginary Experiences.” Nature Communications 15, no. 1: 1002. 10.1038/s41467-024-45065-w PMC10837448 38307834

[brb370393-bib-0018] Biderman, D. , Y. Shir , and L. Mudrik . 2020. “B or 13? Unconscious Top‐Down Contextual Effects at the Categorical but Not the Lexical Level.” Psychological Science 31, no. 6: 663–677. 32384011 10.1177/0956797620915887 PMC7289051

[brb370393-bib-0019] Block, N. 2024. “What Does Decoding From the PFC Reveal About Consciousness?” Trends in Cognitive Sciences 28, no. 9: 804–813. 38862352 10.1016/j.tics.2024.05.004

[brb370393-bib-0020] Boly, M. , M. Massimini , N. Tsuchiya , B. R. Postle , C. Koch , and G. Tononi . 2017. “Are the Neural Correlates of Consciousness in the Front or in the Back of the Cerebral Cortex? Clinical and Neuroimaging Evidence.” Journal of Neuroscience 37, no. 40: 9603–9613. 28978697 10.1523/JNEUROSCI.3218-16.2017 PMC5628406

[brb370393-bib-0021] Bouvier, S. , and A. Treisman . 2010. “Visual Feature Binding Requires Reentry.” Psychological Science 21, no. 2: 200–204. 20424045 10.1177/0956797609357858 PMC3113689

[brb370393-bib-0022] Bracci, S. , and H. P. Op de Beeck . 2023. “Understanding Human Object Vision: A Picture Is Worth a Thousand Representations.” Annual Review of Psychology 74, no. 1: 113–135. 10.1146/annurev-psych-032720-041031 36378917

[brb370393-bib-0023] Brascamp, J. W. , and S. K. Shevell . 2021. “The Certainty of Ambiguity in Visual Neural Representations.” Annual Review of Vision Science 7, no. 1: 465–486. 10.1146/annurev-vision-100419-125929 PMC8687672 34524881

[brb370393-bib-0024] Bruner, J. S. , and A. L. Minturn . 1955. “Perceptual Identification and Perceptual Organization.” Journal of General Psychology 53, no. 1: 21–28.

[brb370393-bib-0025] Capone, C. , C. Lupo , P. Muratore , and P. S. Paolucci . 2023. “Beyond Spiking Networks: The Computational Advantages of Dendritic Amplification and Input Segregation.” Proceedings of the National Academy of Sciences 120, no. 49: e2220743120. 10.1073/pnas.2220743120 PMC10710097 38019856

[brb370393-bib-0026] Chis‐Ciure, R. , L. Melloni , and G. Northoff . 2024. “A Measure Centrality Index for Systematic Empirical Comparison of Consciousness Theories.” Neuroscience & Biobehavioral Reviews 105670. 1–22. 38615851 10.1016/j.neubiorev.2024.105670

[brb370393-bib-0027] Collerton, D. , J. Barnes , N. J. Diederich , et al. 2023. “Understanding Visual Hallucinations: A New Synthesis.” Neuroscience & Biobehavioral Reviews 150: 105208. 37141962 10.1016/j.neubiorev.2023.105208

[brb370393-bib-0028] Crick, F. , and C. Koch . 1990. “Towards a Neurobiological Theory of consciousness.” In Seminars in the Neurosciences, Vol. 2: 263–275.

[brb370393-bib-0029] Evers, K. , M. Farisco , and C. M. A. Pennartz . 2024. “Assessing the Commensurability of Theories of Consciousness: On the Usefulness of Common Denominators in Differentiating, Integrating and Testing Hypotheses.” Consciousness and Cognition 119: 103668. 38417198 10.1016/j.concog.2024.103668

[brb370393-bib-0030] Faivre, N. , J. Dubois , N. Schwartz , and L. Mudrik . 2019. “Imaging Object‐Scene Relations Processing in Visible and Invisible Natural Scenes.” Scientific Reports 9, no. 1: 4567. 30872607 10.1038/s41598-019-38654-z PMC6418099

[brb370393-bib-0031] Filimonov, D. , H. Railo , A. Revonsuo , and M. Koivisto . 2022. “Modality‐Specific and Modality‐General Electrophysiological Correlates of Visual and Auditory Awareness: Evidence From a Bimodal ERP Experiment.” Neuropsychologia 166: 108154. 35016890 10.1016/j.neuropsychologia.2022.108154

[brb370393-bib-0032] Förster, J. , M. Koivisto , and A. Revonsuo . 2020. “ERP and MEG Correlates of Visual Consciousness: The Second Decade.” Consciousness and Cognition 80: 102917. 32193077 10.1016/j.concog.2020.102917

[brb370393-bib-0033] Frith, C. D. , R. Perry , and E. Lumer . 1999. “The Neural Correlates of Conscious Experience: An Experimental Framework.” Trends in Cognitive Sciences 3, no. 3: 105–114. 10322462 10.1016/s1364-6613(99)01281-4

[brb370393-bib-0034] Gazzaniga, M. S. , R. B. Ivry , and G. R. Mangun . 2019. Cognitive Neuroscience: The Biology of the Mind. 5th ed. New York: Norton.

[brb370393-bib-0035] Gelbard‐Sagiv, H. , E. Magidov , H. Sharon , T. Hendler , and Y. Nir . 2018. “Noradrenaline Modulates Visual Perception and Late Visually Evoked Activity.” Current Biology 28, no. 14: 2239–2249. 29983318 10.1016/j.cub.2018.05.051

[brb370393-bib-0036] Gillon, C. J. , J. E. Pina , J. A. Lecoq , et al. 2024. “Responses to Pattern‐Violating Visual Stimuli Evolve Differently Over Days in Somata and Distal Apical Dendrites.” Journal of Neuroscience 44, no. 5:. 10.1523/JNEUROSCI.1009-23.2023 PMC10860604 37989593

[brb370393-bib-0037] Gori, M. , D. Burr , and C. Campus . 2024. “Disambiguating Vision With Sound.” Current Biology 34, no. 6: R235–R236. 38531313 10.1016/j.cub.2024.01.043

[brb370393-bib-0038] Granato, A. , W. A. Phillips , J. M. Schulz , M. Suzuki , and M. E. Larkum . 2024. “Dysfunctions of Cellular Context‐Sensitivity in Neurodevelopmental Learning Disabilities.” Neuroscience & Biobehavioral Reviews 161: 105688. 38670298 10.1016/j.neubiorev.2024.105688

[brb370393-bib-0040] Hamm, J. P. , Y. Shymkiv , S. Han , W. Yang , and R. Yuste . 2021. “Cortical Ensembles Selective for Context.” Proceedings of the National Academy of Sciences 118, no. 14: e2026179118. 10.1073/pnas.2026179118 PMC8040629 33811144

[brb370393-bib-0041] Hansen, M. K. 2024. “Perceiving Affordances and the Problem of Visually Indiscernible Kinds.” Frontiers in Psychology 15: 1388852. 39295750 10.3389/fpsyg.2024.1388852 PMC11409843

[brb370393-bib-0042] Hesselmann, G. , C. A. Kell , E. Eger , and A. Kleinschmidt . 2008. “Spontaneous Local Variations in Ongoing Neural Activity Bias Perceptual Decisions.” Proceedings of the National Academy of Sciences 105, no. 31: 10984–10989. 10.1073/pnas.0712043105 PMC2504783 18664576

[brb370393-bib-0043] Ishizu, T. , and S. Zeki . 2014. “Varieties of Perceptual Instability and Their Neural Correlates.” Neuroimage 91: 203–209. 24486830 10.1016/j.neuroimage.2014.01.040 PMC3985424

[brb370393-bib-0044] Jimenez, M. , S. Grassini , P. R. Montoro , D. Luna , and M. Koivisto . 2018. “Neural Correlates of Visual Awareness at Stimulus Low vs. High‐Levels of Processing.” Neuropsychologia 121: 144–152. 30408463 10.1016/j.neuropsychologia.2018.11.001

[brb370393-bib-0045] Kim, C. Y. , and R. Blake . 2005. “Psychophysical Magic: Rendering the Visible ‘Invisible’.” Trends in Cognitive Sciences 9, no. 8: 381–388. 16006172 10.1016/j.tics.2005.06.012

[brb370393-bib-0046] Klein, C. , J. Hohwy , and T. Bayne . 2020. “Explanation in the Science of Consciousness: From the Neural Correlates of Consciousness (NCCs) to the Difference Makers of Consciousness (DMCs).” Philosophy and the Mind Sciences 1, no. II: 1–20.

[brb370393-bib-0047] Klink, P. C. , R. J. A. van Wezel , and R. van Ee . 2012. “United We Sense, Divided We Fail: Context‐Driven Perception of Ambiguous Visual Stimuli.” Philosophical Transactions of the Royal Society B: Biological Sciences 367, no. 1591: 932–941. 10.1098/rstb.2011.0358 PMC3282309 22371615

[brb370393-bib-0048] Koch, C. 2004. The Quest for Consciousness: A Neurobiological Approach. Englewood, Roberts & Company.

[brb370393-bib-0049] Koch, C. , M. Massimini , M. Boly , and G. Tononi . 2016. “Neural Correlates of Consciousness: Progress and Problems.” Nature Reviews Neuroscience 17, no. 5: 307–321. 27094080 10.1038/nrn.2016.22

[brb370393-bib-0050] Köhler, W. , and P. A. Adams . 1958. “Perception and Attention.” American Journal of Psychology 71, no. 3: 489–503. 13571453

[brb370393-bib-0051] Kozuch, B. 2024. “An Embarrassment of Richnesses: The PFC Isn't the Content NCC.” Neuroscience of Consciousness 2024, no. 1: niae017. 38938921 10.1093/nc/niae017 PMC11210398

[brb370393-bib-0052] Lamme, V. A. 2018. “Challenges for Theories of Consciousness: Seeing or Knowing, the Missing Ingredient and How to Deal With Panpsychism.” Philosophical Transactions of the Royal Society B: Biological Sciences 373, no. 1755: 20170344. 10.1098/rstb.2017.0344 PMC6074090 30061458

[brb370393-bib-0053] Larkum, M. 2013. “A Cellular Mechanism for Cortical Associations: An Organizing Principle for the Cerebral Cortex.” Trends in Neurosciences 36, no. 3: 141–151. 23273272 10.1016/j.tins.2012.11.006

[brb370393-bib-0054] Larkum, M. E. , and W. A. Phillips . 2016. “Does Arousal Enhance Apical Amplification and Disamplification?” Behavioral and Brain Sciences 39: 32–34. 10.1017/S0140525X15001867 28347375

[brb370393-bib-0055] Lepauvre, A. , and L. Melloni . 2021. “The Search for the Neural Correlate of Consciousness: Progress and Challenges.” Philosophy and the Mind Sciences 2: 1–28.

[brb370393-bib-0056] Leticevscaia, O. , T. Brandman , and M. V. Peelen . 2024. “Scene Context and Attention Independently Facilitate MEG Decoding of Object Category.” Vision Research 224: 108484. 39260230 10.1016/j.visres.2024.108484

[brb370393-bib-0057] Long, G. M. , and T. C. Toppino . 2004. “Enduring Interest in Perceptual Ambiguity: Alternating Views of Reversible Figures.” Psychological Bulletin 130, no. 5: 748–768. 15367079 10.1037/0033-2909.130.5.748

[brb370393-bib-0058] MacDonald, J. , S. Andersen , and T. Bachmann . 2000. “Hearing by Eye: How Much Spatial Degradation Can be Tolerated?” Perception 29, no. 10: 1155–1168. 11220208 10.1068/p3020

[brb370393-bib-0059] Maksimov, M. , M. Vaht , J. Harro , and T. Bachmann . 2013. “Can Common Functional Gene Variants Affect Visual Discrimination in Metacontrast Masking?” PLOS ONE 8, no. 1: e55287. 23359627 10.1371/journal.pone.0055287 PMC3554658

[brb370393-bib-0060] Maksimov, M. , M. Vaht , J. Harro , and T. Bachmann . 2015. “Single 5HTR2A‐1438 A/G Nucleotide Polymorphism Affects Performance in a Metacontrast Masking Task: Implications for Vulnerability Testing and Neuromodulation of Pyramidal Cells.” Neuroscience Letters 584: 129–134. 25459290 10.1016/j.neulet.2014.10.015

[brb370393-bib-0061] Maksimov, M. , M. Vaht , C. Murd , J. Harro , and T. Bachmann . 2017. “Variants of TPH2 Interact With Fast Visual Processing as Assessed by Metacontrast.” Neuroreport 28, no. 2: 111–114. 27926628 10.1097/WNR.0000000000000721

[brb370393-bib-0062] Mao, Y. , R. Kanai , C. Ding , T. Bi , and J. Qiu . 2020. “Temporal Variability of Brain Networks Predicts Individual Differences in Bistable Perception.” Neuropsychologia 142: 107426. 32147392 10.1016/j.neuropsychologia.2020.107426

[brb370393-bib-0063] Marek, G. J. , and D. D. Schoepp . 2021. “Cortical Influences of Serotonin and Glutamate on Layer V Pyramidal Neurons.” Progress in Brain Research 261: 341–378. 33785135 10.1016/bs.pbr.2020.11.002

[brb370393-bib-0064] Marvan, T. , and W. A. Phillips . 2024. “Cellular Mechanisms of Cooperative Context‐Sensitive Predictive Inference.” Current Research in Neurobiology 6: 100129. 1–12. 38665363 10.1016/j.crneur.2024.100129 PMC11043869

[brb370393-bib-0065] Marvan, T. , M. Polák , T. Bachmann , and W. A. Phillips . 2021. “Apical Amplification—A Cellular Mechanism of Conscious Perception?” Neuroscience of Consciousness 2021, no. 2: niab036. 34650815 10.1093/nc/niab036 PMC8511476

[brb370393-bib-0066] Mather, M. , D. Clewett , M. Sakaki , and C. W. Harley . 2016. “Norepinephrine Ignites Local Hotspots of Neuronal Excitation: How Arousal Amplifies Selectivity in Perception and Memory.” Behavioral and Brain Sciences 39: e200. 26126507 10.1017/S0140525X15000667 PMC5830137

[brb370393-bib-0067] McGurk, H. , and J. MacDonald . 1976. “Hearing Lips and Seeing Voices.” Nature 264, no. 5588: 746–748. 1012311 10.1038/264746a0

[brb370393-bib-0068] Millman, D. J. , G. K. Ocker , S. Caldejon , et al. 2020. “VIP Interneurons in Mouse Primary Visual Cortex Selectively Enhance Responses to Weak but Specific Stimuli.” eLife 9: e55130. 33108272 10.7554/eLife.55130 PMC7591255

[brb370393-bib-0069] Munn, B. R. , E. J. Müller , J. Aru , et al. 2023. “A Thalamocortical Substrate for Integrated Information via Critical Synchronous Bursting.” Proceedings of the National Academy of Sciences 120, no. 46: e2308670120. 10.1073/pnas.2308670120 PMC10655573 37939085

[brb370393-bib-0070] Munn, B. R. , E. J. Müller , V. Medel , et al. 2023. “Neuronal Connected Burst Cascades Bridge Macroscale Adaptive Signatures Across Arousal States.” Nature Communications 14, no. 1: 6846. 10.1038/s41467-023-42465-2 PMC10611774 37891167

[brb370393-bib-0071] Murray, M. M. , and C. S. Herrmann . 2013. “Illusory Contours: A Window Onto the Neurophysiology of Constructing Perception.” Trends in Cognitive Sciences 17, no. 9: 471–481. 23928336 10.1016/j.tics.2013.07.004

[brb370393-bib-0072] Navajas, J. , H. G. Rey , and R. Quian Quiroga . 2014. “Perceptual and Contextual Awareness: Methodological Considerations in the Search for the Neural Correlates of Consciousness.” Frontiers in Psychology 5: 959. 25221537 10.3389/fpsyg.2014.00959 PMC4148639

[brb370393-bib-0073] Northoff, G. , and V. Lamme . 2020. “Neural Signs and Mechanisms of Consciousness: Is There a Potential Convergence of Theories of Consciousness in Sight?.” Neuroscience & Biobehavioral Reviews 118: 568–587. 32783969 10.1016/j.neubiorev.2020.07.019

[brb370393-bib-0074] Otten, M. , A. K. Seth , and Y. Pinto . 2017. “A Social Bayesian Brain: How Social Knowledge Can Shape Visual Perception.” Brain and Cognition 112: 69–77. 27221986 10.1016/j.bandc.2016.05.002

[brb370393-bib-0076] Palmeri, T. J. , and I. Gauthier . 2004. “Visual Object Understanding.” Nature Reviews Neuroscience 5, no. 4: 291–303. 15034554 10.1038/nrn1364

[brb370393-bib-0077] Pang, Z. , C. B. O'May , B. Choksi , and R. VanRullen . 2021. “Predictive Coding Feedback Results in Perceived Illusory Contours in a Recurrent Neural Network.” Neural Networks 144: 164–175. 34500255 10.1016/j.neunet.2021.08.024

[brb370393-bib-0078] Parr, T. , A. W. Corcoran , K. J. Friston , and J. Hohwy . 2019. “Perceptual Awareness and Active Inference.” Neuroscience of Consciousness 2019, no. 1: niz012. 31528360 10.1093/nc/niz012 PMC6734140

[brb370393-bib-0079] Paßler, M. 2023. “The Exclusionary Approach to Consciousness.” Neuroscience of Consciousness 2023, no. 1: niad022. 37810758 10.1093/nc/niad022 PMC10553408

[brb370393-bib-0080] Phillips, W. A. 2017. “Cognitive Functions of Intracellular Mechanisms for Contextual Amplification.” Brain and Cognition 112: 39–53. 26428863 10.1016/j.bandc.2015.09.005

[brb370393-bib-0081] Phillips, W. A. 2023. The Cooperative Neuron: Cellular Foundations of Mental Life. Oxford University Press.

[brb370393-bib-0082] Phillips, W. A. , T. Bachmann , and J. F. Storm . 2018. “Apical Function in Neocortical Pyramidal Cells: A Common Pathway by Which General Anesthetics Can Affect Mental state.” Frontiers in Neural Circuits 12: 50. 30013465 10.3389/fncir.2018.00050 PMC6036169

[brb370393-bib-0083] Phillips, W. A. , T. Bachmann , M. W. Spratling , L. Muckli , L. S. Petro , and T. Zolnik . 2024. “Cellular Psychology: Relating Cognition to Context‐Sensitive Pyramidal Cells.” Trends in Cognitive Sciences 29, no. 1: 28–40. 10.1016/j.tics.2024.09.002. 39353837

[brb370393-bib-0084] Pitts, M. A. , W. J. Gavin , and J. L. Nerger . 2008. “Early Top‐Down Influences on Bistable Perception Revealed by Event‐Related Potentials.” Brain and Cognition 67, no. 1: 11–24. 18155339 10.1016/j.bandc.2007.10.004

[brb370393-bib-0085] Pitts, M. A. , S. Metzler , and S. A. Hillyard . 2014. “Isolating Neural Correlates of Conscious Perception From Neural Correlates of Reporting One's Perception.” Frontiers in Psychology 5: 1078. 25339922 10.3389/fpsyg.2014.01078 PMC4189413

[brb370393-bib-0086] Promet, L. , and T. Bachmann . 2024. “A Comparative Analysis of Empirical Theories of Consciousness.” Psychology of Consciousness: Theory, Research, and Practice 11, no. 4: 493–526.

[brb370393-bib-0120] Rassi, E. , A. Wutz , N. Müller‐Voggel , and N. Weisz . 2019. “Prestimulus feedback connectivity biases the content of visual experiences.” Proceedings of the National Academy of Sciences 116, no. 32: 16056–16061. 10.1073/pnas.1817317116 PMC6689959 31332019

[brb370393-bib-0087] Quiroga, R. 2013. “Gnostic Cells in the 21st Century.” Acta Neurobiologiae Experimentalis 73, no. 4: 463–471. 24457638 10.55782/ane-2013-1952

[brb370393-bib-0088] Rossel, P. , C. Peyrin , A. Roux‐Sibilon , and L. Kauffmann . 2022. “It Makes Sense, so I See It Better! Contextual Information About the Visual Environment Increases Its Perceived Sharpness.” Journal of Experimental Psychology: Human Perception and Performance 48, no. 4: 331. 35130017 10.1037/xhp0000993

[brb370393-bib-0089] Rossel, P. , C. Peyrin , and L. Kauffmann . 2023. “Subjective Perception of Objects Depends on the Interaction Between the Validity of Context‐Based Expectations and Signal Reliability.” Vision Research 206: 108191. 36773476 10.1016/j.visres.2023.108191

[brb370393-bib-0090] Seghier, M. L. , and P. Vuilleumier . 2006. “Functional Neuroimaging Findings on the Human Perception of Illusory Contours.” Neuroscience & Biobehavioral Reviews 30, no. 5: 595–612. 16457887 10.1016/j.neubiorev.2005.11.002

[brb370393-bib-0091] Sen, S. , S. N. Daimi , K. Watanabe , K. Takahashi , J. Bhattacharya , and G. Saha . 2020. “Switch or Stay? Automatic Classification of Internal Mental States in Bistable Perception.” Cognitive Neurodynamics 14: 95–113. 32015769 10.1007/s11571-019-09548-7 PMC6973829

[brb370393-bib-0092] Shin, H. , and H. Adesnik . 2024. “Functional Roles of Cortical Inhibitory Interneurons.” Cerebral Cortex and Thalamus 72: 72–80.

[brb370393-bib-0093] Shin, J. N. , G. Doron , and M. E. Larkum . 2021. “Memories Off the Top of Your Head.” Science 374, no. 6567: 538–539. 34709915 10.1126/science.abk1859 PMC7612398

[brb370393-bib-0094] Shine, J. M. 2023. “Neuromodulatory Control of Complex Adaptive Dynamics in the Brain.” Interface Focus 13: 20220079. 37065268 10.1098/rsfs.2022.0079 PMC10102735

[brb370393-bib-0095] Shine, J. M. , E. J. Müller , B. Munn , J. Cabral , R. J. Moran , and M. Breakspear . 2021. “Computational Models Link Cellular Mechanisms of Neuromodulation to Large‐Scale Neural Dynamics.” Nature Neuroscience 24, no. 6: 765–776. 33958801 10.1038/s41593-021-00824-6

[brb370393-bib-0096] Shine, J. M. , L. D. Lewis , D. D. Garrett , and K. Hwang . 2023. “The Impact of the Human Thalamus on Brain‐Wide Information Processing.” Nature Reviews Neuroscience 24: 416–430. 37237103 10.1038/s41583-023-00701-0 PMC10970713

[brb370393-bib-0097] Storm, J. F. , P. C. Klink , J. Aru , et al. 2024. “An Integrative, Multiscale View on Neural Theories of Consciousness.” Neuron 112, no. 10: 1531–1552. 38447578 10.1016/j.neuron.2024.02.004

[brb370393-bib-0098] Suzuki, M. , and M. E. Larkum . 2020. “General Anesthesia Decouples Cortical Pyramidal Neurons.” Cell 180, no. 4: 666–676. 32084339 10.1016/j.cell.2020.01.024

[brb370393-bib-0099] Takahashi, N. , C. Ebner , J. Sigl‐Glöckner , S. Moberg , S. Nierwetberg , and M. E. Larkum . 2020. “Active Dendritic Currents Gate Descending Cortical Outputs in Perception.” Nature Neuroscience 23, no. 10: 1277–1285. 32747790 10.1038/s41593-020-0677-8

[brb370393-bib-0100] Takahashi, N. , T. G. Oertner , P. Hegemann , and M. E. Larkum . 2016. “Active Cortical Dendrites Modulate Perception.” Science 354, no. 6319: 1587–1590. 28008068 10.1126/science.aah6066

[brb370393-bib-0101] Tal, A. , M. Sar‐Shalom , T. Krawitz , D. Biderman , and L. Mudrik . 2024. “Awareness Is Needed for Contextual Effects in Ambiguous Object Recognition.” Cortex 173: 49–60. 38367591 10.1016/j.cortex.2024.01.003

[brb370393-bib-0102] Treisman, A. 1987. “Properties, Parts, and Objects.” In Handbook of Perception and Human Performance, edited by K. R. Boff , I. Kaufmann and F. P. Thomas , Vol. 2, 35–31 – 35–70: Wiley.

[brb370393-bib-0103] Tulver, K. , J. Aru , R. Rutiku , and T. Bachmann . 2019. “Individual Differences in the Effects of Priors on Perception: A Multi‐Paradigm Approach.” Cognition 187: 167–177. 30877848 10.1016/j.cognition.2019.03.008

[brb370393-bib-0104] Van Geert, E. , and J. Wagemans . 2024. “Prägnanz in Visual Perception.” Psychonomic Bulletin & Review 31, no. 2: 541–567. 37787874 10.3758/s13423-023-02344-9 PMC11061049

[brb370393-bib-0105] Vetik, S. , K. Tulver , D. Lints , and T. Bachmann . 2020. “Among the Two Kinds of Metacognitive Evaluation, Only One Is Predictive of Illusory Object Perception.” Perception 49, no. 10: 1043–1056. 32903160 10.1177/0301006620954322

[brb370393-bib-0107] Vollenweider, F. X. , and K. H. Preller . 2020. “Psychedelic Drugs: Neurobiology and Potential for Treatment of Psychiatric Disorders.” Nature Reviews Neuroscience 21, no. 11: 611–624. 32929261 10.1038/s41583-020-0367-2

[brb370393-bib-0108] von Gal, A. , M. Boccia , R. Nori , P. Verde , A. M. Giannini , and L. Piccardi . 2023. “Neural Networks Underlying Visual Illusions: An Activation Likelihood Estimation Meta‐Analysis.” Neuroimage 279: 120335. 37591478 10.1016/j.neuroimage.2023.120335

[brb370393-bib-0121] Wang, X. , N. Sang , L. Hao , Y. Zhang , T. Bi , and J. Qiu . 2017. “Category selectivity of human visual cortex in perception of rubin face–vase illusion.” Frontiers in psychology 8: 1543. 28955269 10.3389/fpsyg.2017.01543 PMC5600935

[brb370393-bib-0109] Wagemans, J. , ed. 2015. The Oxford Handbook of Perceptual Organization. OUP Oxford.

[brb370393-bib-0110] Wagemans, J. , J. H. Elder , M. Kubovy , et al. 2012. “A Century of Gestalt Psychology in Visual Perception: I. Perceptual Grouping and Figure–Ground Organization.” Psychological Bulletin 138, no. 6: 1172. 22845751 10.1037/a0029333 PMC3482144

[brb370393-bib-0111] Watanabe, T. 2021. “Causal Roles of Prefrontal Cortex During Spontaneous Perceptual Switching Are Determined by Brain state Dynamics.” eLife 10: e69079. 34713803 10.7554/eLife.69079 PMC8631941

[brb370393-bib-0112] Weilnhammer, V. A. , M. Fritsch , M. Chikermane , et al. 2021. “An Active Role of Inferior Frontal Cortex in Conscious Experience.” Current Biology 31, no. 13: 2868–2880. 33989530 10.1016/j.cub.2021.04.043

[brb370393-bib-0114] Werner, J. S. , and L. M. Chalupa , eds. 2004. The Visual Neurosciences. Mit Press.

[brb370393-bib-0115] Whyte, C. J. , M. J. Redinbaugh , J. M. Shine , and Y. B. Saalmann . 2024. “Thalamic Contributions to the State and Contents of Consciousness.” Neuron 112, no. 10: 1611–1625. 38754373 10.1016/j.neuron.2024.04.019 PMC11537458

[brb370393-bib-0116] Yan, Y. , J. Zhan , O. Garrod , X. Cui , R. A. Ince , and P. G. Schyns . 2023. “Strength of Predicted Information Content in the Brain Biases Decision Behavior.” Current Biology 33, no. 24: 5505–5514. 38065096 10.1016/j.cub.2023.10.042

[brb370393-bib-0117] Yaron, I. , L. Melloni , M. Pitts , and L. Mudrik . 2022. “The ConTraSt Database for Analysing and Comparing Empirical Studies of Consciousness Theories.” Nature Human Behaviour 6, no. 4: 593–604. 10.1038/s41562-021-01284-5 35190711

[brb370393-bib-0118] Zimmerman, A. W. , M. E. Blue , M. V. Johnston , C. B. Moloney , and C. F. Hohmann . 2008. “Serotonin Dysfunction in autism.” In Autism: Current Theories and Evidence, A. W. Zimmerman (ed.), Autism, Humana Press, Totowa, NJ 2008. 111–132.

